# Wnt/ERK/CDK4/6 activation in the partial EMT state coordinates mammary cancer stemness with self-renewal and inhibition of differentiation

**DOI:** 10.1038/s41416-025-03074-6

**Published:** 2025-06-24

**Authors:** Huizhi Liang, Outhiriaradjou Benard, Viney Kumar, Anthony Griffen, Zuen Ren, Kalaiselvi Sivalingam, Jingli Wang, Elena de Simone Benito, Xusheng Zhang, Jinghang Zhang, Kimita Suyama, Lindsay M. LaFave, Larry Norton, Rachel B. Hazan

**Affiliations:** 1 Department of Pathology, Albert Einstein College of Medicine, Bronx, NY USA; 2 Department of Cell Biology, Albert Einstein College of Medicine, Bronx, NY USA; 3 Center for Cancer Research, Massachusetts General Hospital and Harvard Medical School, Boston, MA USA; 4 Francisco de Vitoria University, Madrid, Spain; 5 Computational Genomics Core, Albert Einstein College of Medicine, Bronx, NY USA; 6 Department of Microbiology and Immunology, Albert Einstein College of Medicine, Bronx, NY USA; 7 Department of Medicine, Memorial Sloan-Kettering Cancer Center, New York, NY USA; 8 Cancer Dormancy Institute, Albert Einstein College of Medicine, Bronx, New York, NY USA

**Keywords:** Cancer stem cells, Cancer

## Abstract

**Background:**

The hybrid EMT state is a key driver of tumour regenerative and metastatic potential; however, the mechanism whereby this programme regulates tumour stemness with respect to self-renewal and differentiation remains unclear.

**Methods:**

We isolated epithelial/mesenchymal (E/M) (CD104^high^CD44^high^) and mesenchymal (M) (CD104^low^ CD44^high^) subpopulations from basal-like breast cancer cell lines. These were assayed for tumour-initiating potential and organoid-forming ability, as well as for transcriptional regulators of the hybrid EMT state by RNA and ATAC-sequencing, and their regulation by the Wnt/ERK/CDK4/6 signalling pathway.

**Results:**

E/M cells were endowed with organoid-forming ability as well as by tumour-initiating and metastatic potential relative to M cells. Interestingly, Wnt3a stimulates *transient* ERK/CDK4/6 activation in E/M cells, thereby upregulating FOXC2, and in turn TAp63 and ΔNp63, which support the hybrid state. In parallel, ERK/CDK4/6 activates S-phase and FOXM1, thereby promoting self-renewal. Remarkably, transient ERK activation by Wnt3a deactivates EGFR, thus preventing sustained ERK phosphorylation from causing E/M differentiation. Consistently, ERK/CDK4/6 drug perturbation in E/M cells suppressed FOXC2/p63, FOXM1, self-renewal, organoid formation and mammary tumour growth via epithelial differentiation.

**Conclusions:**

These findings unravelled a mechanism whereby the hybrid EMT state regulates stemness, self-renewal and differentiation via transient Wnt/ERK/CDK4/6 activation, which can be leveraged for cancer stem cell therapy.

## Background

Metastasis is the major cause of morbidity in breast cancer patients [1]. This process begins with the exit of carcinoma cells from the primary tumour, invasion into the surrounding stroma and vasculature, survival in circulation, extravasation, seeding at distant organs and growth into overt metastases [1]. The epithelial-to-mesenchymal transition (EMT) is a pivotal process in metastasis that generates aggressive tumour cells with cancer stem cell properties [2–4]. These CSCs consist of a minor subpopulation that grows and self-renews to maintain the CSC pool while giving rise to differentiated progenitors, making up the bulk of the tumour. EMT is induced by cytokines as TGFβ, Wnt, Jagged, IL6, etc., that may be released by tumour cells and/or cancer-associated stromal or immune cells [2–4]. This results in the expression of EMT transcription factors such as Twist, Slug, Snail, Zeb1/2, and FOXC2 among others, which repress epithelial differentiation and shift cells towards a mesenchymal phenotype [3, 5].

Cumulative evidence has shifted our view on EMT, pointing to a dynamic process capable of transiting carcinoma cells from an epithelial to a hybrid epithelial/mesenchymal (E/M) or mesenchymal (M) state. Moreover, cells undergoing partial EMT may adopt a continuum of E/M states, further exacerbating tumour heterogeneity, resulting in chemoresistance and metastasis [6–10]. Using the well-established epithelial CD104 (β4 integrin) and mesenchymal CD44 cell surface markers, we isolated E/M and M cells from basal-like breast cancer cell lines. E/M cells (CD104^high^CD44^high^) were shown to be endowed with tumour-initiating and metastatic potential relative to cells residing in E (CD104^high^CD44^low^) or M (CD104^low^CD44^high^) state [11, 12]. Moreover, E/M cells were enriched in Snail and Wnt/β-catenin signalling as compared to M cells, which upregulate Zeb-1 and non-canonical Wnt signalling [12], underscoring the role of Wnt pathways in regulating the EMT spectrum.

Wnt/β-catenin signalling is a powerful driver of stemness in normal and malignant cells [13] which is regulated by transcription factor T-cell factor (TCF) including TCF1, that is encoded by the TCF7 gene [14]. Canonical Wnt signalling is initiated by the binding of Wnt ligands to the Frizzled/LRP5/6 complex, resulting in uncoupling of β-catenin from the destruction complex, and translocation to the nucleus, leading to TCF/LEF transcriptional activation of genes driving cancer stemness and self-renewal [13, 14]. A naturally occurring truncated form of TCF1, lacking the β-catenin binding site, acts as a “dominant-negative” of TCF1 and as a repressor of the pathway [14]. By contrast, non-canonical Wnt signalling, transduced by Wnt/PCP, Wnt/Ca^2+^ or Wnt/RTK pathways, regulates cancer cell migration or invasion, but also stemness [15].

The Wnt/β-catenin pathway activates cancer stem cell maintenance via promotion of EMT transcription factors such as Snail and FOXC2. Interestingly, FOXC2 promotes tumour aggressiveness and poor prognosis in basal-like triple-negative breast cancers via activation of EMT, stemness and drug resistance [16–18]. Likewise, the transcription factor, FOXM1, plays a critical role in TNBC by driving cell proliferation, invasion and drug resistance via direct regulation of genes involved in cell cycle control, DNA damage repair and EMT [19–22]. Thus, we speculate that coincident expression of FOXC2 and FOXM1 in basal-like breast cancer cells contributes to both the maintenance and self-renewal of carcinoma cells undergoing a partial EMT, thereby supporting the stemness and aggressiveness of the TNBC subtype.

Using E/M (CD104^high^CD44^high^) and M (CD104^low^ CD44^high^) subpopulations isolated from MDA-MB-468 and HCC1143 basal-like breast cancer cell lines, we uncovered an upregulation of TCF1 and FOXC2 in E/M relative to M cells, which was in turn able to convert M into E/M cells. Interestingly, Wnt3a caused *transient* ERK/CDK4/6 activation in E/M cells, resulting in FOXC2 upregulation, which stimulates the expression of TAp63 and ΔNp63 isoforms, which together support the hybrid EMT state. In parallel, ERK/CDK4/6 stimulates S-phase and FOXM1 activation in E/M cells, thereby driving self-renewal. Remarkably, transient Wnt/ERK activation in E/M cells inactivates EGFR, which prevents EGF from promoting sustained ERK phosphorylation that causes E/M to M differentiation. Finally, these findings were supported by ATAC-seq, unveiling genome-wide open chromatin accessibility at TCF7, FOXC2, and FOXM1 motifs in E/M cells relative to M cells, which was reversed by ERK/CDK4/6 drug inhibition and was accompanied by suppression of mammary tumour growth and organoid formation in vitro and ex vivo. Collectively, these results highlight an intricate programme governed by TCF1/Wnt signalling which activates ERK/CDK4/6, thus integrating cancer stemness with self-renewal and inhibition of differentiation.

## Methods

### Cell lines and culture

MCF10A, MDA-MB-468, HCC1143, and HEK293T were obtained from the American Type Culture Collection (ATCC, Manassas, VA, USA). MCF10A cells were grown in MEGM media with the growth kit provided by Lonza Corporation: MEGM kit (Lonza, Cat# CC-3150). 1% Pen–Strep and 100 ng/ml of cholera toxin (Sigma) were added to the growth medium. MDA-MB-468 and HCC1143 cells were grown in RPMI 1640 (Gibco Cat# 11875093) supplemented with 10% FBS and 1% Pen–Strep (Gibco Cat# 15140122). HEK293T cells were grown in Dulbecco’s Modified Eagle Medium (Gibco Cat# 11965092) supplemented with 10% FBS and 1% Pen–Strep. L-Wnt3a cell line used to secrete Wnt3a in culture was a gift from Dr. Stuart Aaronson (Mount Sinai School of Medicine, NY) and grown in DMEM supplemented with 10% FBS and 1% Pen–Strep. Authentication of the cell lines was performed using the ATCC method described in https://www.atcc.org/Services/Testing%20Services/Cell%20Authentication%20Testing%20Service.aspx.

### Constructs of human TCF7, dnTCF7, and FOXC2 overexpression

Plasmid pENTR221-TCF7, which bears the human TCF7 open-reading frame, was obtained from Addgene (Plasmid #: 79498). The open-reading frame of human TCF7 was subcloned by PCR into EcoRI/Xbal restriction sites of lentiviral expression vector pLVX-puro (Clontech), using forward primer: 5’-CTGGAATTCTGCAGATCGGCCACCATGTAT-3’, and reverse primer: 5’-TTATCTAGAGCACTGTCATCGGAAGGAACGG-3’. The open-reading frame of dnTCF7 was subcloned from the same plasmid by designing the primers excluding the β-catenin binding site. The cloned dnTCF7 inserts were ligated with vector plasmid pLVX-puro (Clontech) at Xhol/Xbal restriction enzyme sites using a forward primer with added His-tag sequences: 5’-TATCTCGAGATGCATCATCATCATCATCACGGCGCGGCAGGCGGCGCAGGGAT-3’, and the reverse primer was the same as used in TCF7 subcloning.

pBabe human FOXC2 was a gift from Bob Weinberg (Addgene plasmid # 15535; RRID:Addgene_15535) Phoenix-alpha cells seeded at 2 × 10^6^ cells in 10-cm plates were transfected with 10 μg pBabe-FOXC2 and 1.2 μg VSV viral gene in the presence of Fugene transfection reagent. After 48 h, the supernatant was collected and used for transfection.

### RNAi interference, shRNA, and CRISPR/Cas9

FOXC2 siRNA was purchased from Santa Cruz Biotechnology and transfected using Lipofactamin 3000^TM^ kit from Invitrogen. Lentiviral mission shRNA clones against human TCF1 (TRCN0000021674) was purchased from Sigma. ShRNA lentiviral vector against human TCF1 and a control non-targeting shRNA were packaged in HEK293T cells and infected into cells. Briefly, 1 × 10^5^ cells/well of MDA-MB-468/EM cells were seeded in a 12-well plate the day before transduction. The following day, 250 μl of viral supernatant containing 10 μg/ml polybrene was added to cells and incubated for 1 h. Then, 10% FBS/RPMI without antibiotics was added to cells for 24 h. Cells were expanded into a 10-cm dish with selective antibiotics.

pLenti-CRISPR-V2 containing either TAp63 sgRNA: 5’-TTTGTCGCACCATCTTCTGA-3’; or ΔNp63 sgRNAs 5’-TACCTCACTAAATTGAGTCT-3’; 5’-TTCATATTGTAAGGGTCTCG-3’ were obtained from the Gene modification shared core facility at Albert Einstein College of Medicine. Briefly, 3 × 10^6^ of 293T cells was transfected with 12 μg pLenti-CRISPR-V2 containing either TAp63 or ΔNp63 sgRNA in the presence of lentiviral packaging genes 0.6 μg TAT, REV, GAG/POL and 1.2 μg of VSV-G. The supernatant was collected after 48 h and centrifuged at 2000 rpm for 10 min, filtered through 0.45-μm pores and stored at −80 °C for subsequent transfection.

### Flow cytometry

Cells were trypsinized, counted and adjusted to <10 × 10^6^ cells in 1 ml of 2% FBS/PBS. Human TruStain FcX^TM^ (Biolegend, Cat# 422302) was used to block Fc Receptors to reduce non-specific binding. Zombie Violet^TM^ from Biolegend was used to distinguish live and dead cells. Cells were stained with anti-CD44-APC and anti-CD104-PE, or anti-CD24-PE at 1:100 dilution for 45 min at room temperature in the dark before being sorted by ThermoFisher Bigfoot Cell Sorter or analysed by Cytek Aurora. The sorted singlet cells were then collected and cultured at 5% CO_2_ and 37 °C for further analysis.

### Antibodies

Antibodies against TCF1, p63, FOXC2, CD104, CD44, TCF4, Notch1, Zeb1, Nanog, ERK, Frizzled 7, p107, p130, Bim and ALDH1, were from Santa Cruz Biotechnology. CD104-PE and CD44-APC were from Biolegend. E-cadherin and N-cadherin were from BD Biosciences. Snail, Slug, Vimentin, p-EGFR (Y1173), EGFR, p-AKT(Ser374), AKT, p-ERK1/2 (Thr202/Tyr204), p-Rb (Ser780), PCNA, Cleaved-PARP were from Cell Signaling Technology. SOX9 was from Millipore-Sigma. FOXM1 and β-actin were from Proteintech. p-FOXM1(Ser35) was from MyBiosource. HRP-conjugated anti-mouse and anti-rabbit secondary antibodies were from Jackson Laboratories. FITC or TRITC-coupled secondary antibodies (Alexa Fluor 594 goat anti-rabbit, Alexa Fluor 488 goat anti-mouse, Alexa Fluor 633 donkey anti-rat, Alexa Fluor 488 donkey anti-rat, Alexa Fluor 568 donkey anti-goat) were from Invitrogen.

### Ligands and inhibitors

Wnt3a conditioned media (CM) was prepared from L-Wnt3a cells. L-Wnt3a cells were cultured in a 10-cm Petri dish with 10 ml DMEM medium containing 10% foetal bovine serum until the cells reached 60% confluency. Then, substitute with 10 ml fresh culture medium without antibiotics and culture for another 2 days. Collect and filter (0.2-μm pore) the Wnt3a-conditioned media and store at −20 °C. For Wnt3a activation, mix 1-part Wnt3a conditioned media and 1-part regular media and add to cultured cells. Wnt3a human recombinant protein was obtained from R&D Systems. Human recombinant EGF was from PeproTech (Cat# AF-100-15), FGF was from Biotechne R&D systems (Cat# 3718-FB-025), insulin was from Santa Cruz Biotechnology (Cat# sc-360248), heparin and hydrocortisone were purchased from Sigma (Cat# H3393; Cat# H0888). The MEK1 inhibitor, PD0325901 (PD901), was from Selleckchem, dissolved in DMSO and stored in −20 °C. The cells were treated with 1 μg/ml of PD0325901 (PD901) for 24 h before protein extraction. Palbociclib or Abemaciclib was from MedChemExpress, dissolved in DMSO and stored in −20 °C. ICG001 was from Selleckchem (Cat # S2662), dissolved in DMSO and stored at −20 °C.

### Protein extraction and immunoblotting

Cells or tissues were lysed in RIPA buffer (ThermoFisher) supplemented with 0.5% Triton X-100, protease and phosphatase inhibitors. Protein lysate concentration was measured by and adjusted to a final concentration of 1 μg/μl with 4× sample buffer. In total, 30 μg protein was loaded onto 7–12% SDS-polyacrylamide gels and transferred to Immobilon membranes. Blots were probed overnight at 4 °C with indicated antibodies and developed by chemiluminescence (Perkin Elmer or Amersham).

### Immunofluorescence staining on cells and paraffin-embedded tissue sections

Cells seeded on coverslips were fixed in 3.7% paraformaldehyde, permeabilised in 0.01% Triton X-100, washed in PBS, and blocked in 5% goat serum/2% BSA/PBS for 1 h. Cells were incubated with primary antibody in blocking buffer for 1 h, washed, and incubated with 1:2000 Alexa Fluor secondary antibody in 2% BSA/PBS for 1.5 h. Cells were then washed in 0.5% BSA/PBS and stained with DAPI.

For tissue immunofluorescence, formalin-fixed paraffin-embedded tumour sections were baked at 55 °C for 1 h, deparaffinized in xylene, and rehydrated in a series of 100% ethanol, 95% ethanol, and distilled water. Antigen retrieval was performed using 1× antigen retrieval solution (Sigma, pH 6.0). Tissues were then incubated with primary antibody in 5% donkey serum, 2% BSA, 0.5%TX-100 in TBS, followed by Alexa Fluor secondary antibody incubation for 1.5 h at room temperature, washed and mounted with mounting media containing DAPI (SouthernBiotech).

### Quantification of tissue immunofluorescence staining

Quantification of immunofluorescence staining was performed using ImageJ software to analyse different regions of the tumour. Specific markers were used to identify distinct cellular populations, and fluorescence intensity measurements were obtained for each marker. Regions of interest (ROIs) were carefully selected to ensure an accurate representation of tumour heterogeneity. The fluorescence signals were quantified using standardised thresholding methods to minimise variability. The resulting quantification data were then subjected to statistical analysis to compare marker expression across different tumour regions, facilitating an objective evaluation of spatial variations in protein expression.

### TOP/FOP flash assay

Cells were plated in 24-well plates in duplicates. Reporter plasmid TOP-Flash or FOP-Flash, which contain three optimal copies of the TCF/LEF-binding site (TOPFlash), or mutated copies of the TCF/LEF-binding site (FOPFlash) upstream of a minimal thymidine kinase promoter directing transcription of a luciferase gene were used. (TOPFlash) or (FOPFlash; 0.5 μg) together with a Renilla luciferase plasmid (0.1 μg) were transfected using Lipofectamine LTX (Invitrogen) according to the manufacturer’s protocol. Transfection was done soon after plating the cells in suspension. To activate the Wnt signalling following 24 h of transfection, Wnt3a-conditioned media with or without 10 μmol/L ICG001 (Selleck Chemicals) were added. Cell lysates were obtained using the lysis buffer provided in the Dual Luciferase Assay Kit (Promega, Cat#E1910). Firefly and Renilla luciferase readings were recorded 48 h post-transfection using Luminometer (Promega). The firefly luciferase activity is then normalised to the Renilla luciferase activity. The fold increase in TOP-Flash activity compared with FOP-Flash was plotted as mean ± SEM of triplicate tests and validated by *t* test, *P* < 0.05.

### Transwell EGF chemotaxis

EGF chemotaxis of MCF10A cells, single-cell suspension of 5 × 10^4^ cells were plated in the top chambers in the transparent cell culture inserts (PET membrane with 8.0-μm pore size) (Falcon, Cat# 353097) in the culture medium (5% horse serum, 10 μg/ml insulin, 0.5 μg/ml hydrocortisone and 100 ng/ml cholera toxin in DMEM/F12 medium) and were allowed to migrate towards medium supplemented with 20 ng/ml EGF for 24 h. The cells migrated to the bottom of the membrane were stained with 0.5% crystal violet/1% formalin/20% methanol for 10 min. Three biological experimental repeats were performed, two pictures/well were taken. Migrated cells were then quantified by ImageJ. Statistical significance was determined by unpaired *t* test, *P* < 0.05 was set for significance.

### Mammosphere formation

Cells were trypsinized, counted and resuspended in a single-cell suspension in DMEM/F12 media (Gibco Cat# 11330032) supplemented with 10 ng/ml FGF2, 4 μg/ml Heparin, 20 ng/ml EGF, 5 μg/ml Insulin and 5 μg/ml hydrocortisone. Overall, 1 × 10^5^ cells were seeded in 60-mm low-attachment plate, and incubated for 5–7 days in a 5% CO_2_/37 °C incubator. Mammospheres were counted in triplicates in a 96-well plate. Mammosphere-forming efficiency (%) was calculated as: (Number of mammospheres per well/number of cells seeded per well) × 100. Statistical analysis was performed using unpaired *t* test, *P* < 0.05 was set for significance.

### BrdU labelling

Cells were seeded at 3 × 10^5^ cells in six-well plates with coverslips. The following day, cells were replaced in 0.2% FBS/RPMI containing 10 μM BrdU and incubated at 37 °C for 24 h in the absence and presence of inhibitors. On the third day, cells were fixed in 3.7% Formalin for 10 min after activation with Wnt3a and permeabilised with Triton-100. For DNA hydrolysis, cells were treated with 2 N HCl for 30 min at RT and neutralised with 0.1 M Sodium Borate pH 8.5 for 30 min. Cells were blocked with 2% BSA in PBS for 1 h and then followed the standard immunostaining protocol. Four to six pictures were taken from each glass coverslips, and BrdU+ cells were counted. Statistical analysis was performed by One-way ANOVA, *P* < 0.05.

### Animal studies

Mice were housed and maintained by the Animal Studies Institute at the Albert Einstein College of Medicine. All animal protocols were reviewed and approved by the Institute for Animal Studies, and all procedures were conducted in accordance with the Institutional Animal Care and Use Committee (IACUC) guidelines. Xenograft models were generated by injection of MDA-MB-468/EM vs. M, MDA-MB-468 CT vs. MDA-MB-468/TCF1OE cells into female athymic nude mice or female NSG mice when indicated. All mice were obtained from Jackson Laboratories. When indicated, NOD scid gamma (NSG) mice (005557, Jackson Laboratory) were used.

### Tumour growth kinetics

The tumour growth curve was determined by measuring tumour size twice a week using a calliper. Tumour volume was calculated using the following formula: tumour volume = shorter diameter^2^ × longer diameter/2. Mice (five per group for MDA-MB-468/EM vs. M; MDA-MB-468 CT vs. MDA-MB-468/TCF1OE) were sacrificed at the endpoint. Data are displayed as mean tumour volume ± SEM. Statistical analyses were performed comparing individual time points by unpaired t-test, and significant differences were established as *P* value < 0.05.

### Limiting dilution assay

For the limiting dilution study, three groups of NSG female mice (*n* = 3 per group) were injected in the inguinal mammary fat pad with varying numbers of E/M (CD44^high^/CD104^high^) cells: 1 × 10⁶, 5 × 10⁵, or 2.5 × 10⁵ cells, suspended in 200 μL of 1× PBS containing 25% Matrigel. To establish mammary xenografts, the contralateral mammary fat pad of each mouse was injected with the same respective number of M (CD44^high^/CD104^low^) cells, isolated from MDA-MB-468 bulk cell line. Tumour growth was monitored from the onset of palpable tumour formation, and the experiment was concluded once any tumour nodule reached or exceeded 1 cm in diameter.

### Drug efficacy study in a mammary xenograft model

For the drug efficacy study, two groups of NSG female mice (vehicle control and treatment, *n* = 3 per group) were injected bilaterally in the inguinal mammary fat pads with 1 × 10⁶ E/M (CD44^high^/CD104^high^) cells suspended in 200 μL of 1× PBS containing 25% Matrigel to generate mammary xenografts. Upon visible of palpable tumours, the treatment group received oral administration of Abemaciclib (50 mg/kg body weight), and PD0325901 (20 mg/kg body weight), for 2 weeks (5 days per week with a 2-day pause in between 2 weeks). Tumour size was measured at regular intervals in both vehicle-treated and drug-treated groups to evaluate therapeutic response. The experiment was terminated once any tumour nodule reached approximately 1 cm in diameter. Excised tumours from both groups were used for organoid formation (ex vivo). These organoids were then treated in vitro with individual drugs and a combination of both drugs to assess their effect.

### Lung metastasis

Mice bearing MDA-MB-468/EM vs. M, and MDA-MB-468 CT vs. TCF1OE mammary tumours were removed at a similar size by survival surgery and sacrificed at 3–4 months post-surgery. Lung tissues were then fixed with 10% formalin for 24 h and paraffin-embedded. Paraffin blocks were cut by a microtome (Leica Microsystems) to generate at least three serial sections at 5 μm in thickness with at least 100 μm intervals between each section. H&E staining was done on the sections by the shared core facility. Lung metastatic foci were counted, and data were displayed as mean foci number ± SEM. Statistical analysis was performed using the unpaired *t* test, and significance was determined at *P* < 0.05.

### Organoid formation

Primary mammary tumours excised from athymic nude mice were minced and digested in (300 Unit/ml Collagenase, type III, 10 μg/ml DNase I, 100 Unit/ml Hyaluronidase, and 5 μM of ROCK inhibitor Y-27632 in DMEM/F12) at 37 °C for 2 h with rotation. After red blood cell lysis was completed, tissues were incubated with 0.05% trypsin-EDTA at 37 °C for 5 min, following a second round of digestion with (1 unit/ml Dispase and 10 μg/ml DNase I in DMEM/F12) at the same condition. Live cells were counted using a Hemocytometer by mixing cell suspension with 0.4% trypan blue. In total, 1 × 10^5^ cells were seeded in a six-well low-attachment plate in organoid culture medium which has a final concentration of 5% Matrigel, 5% FBS, 10 ng/ml EGF, 20 ng/ml FGF2, 4 μg/ml Heparin, and 5 μM Y-27632 in Advanced DMEM/F12 supplemented with 2 mM GlutMAX and 1% Pen–strep. Organoids were counted after 4–7 days in triplicate in 96-well plates. The diameter of each organoid was measured using ImageJ by calculating the average of two measurements. Organoid-forming efficiency (%) was calculated as: (number of organoids per well/number of cells seeded per well) × 100. Statistical analysis was performed using either unpaired *t* test, or one-way ANOVA, and significance was determined at *P* < 0.05.

### Real-time qRT-PCR

RNA was isolated using a tissue/cell total RNA mini kit from EZ BioResearch following the manufacturer’s protocol. To generate cDNA, 1 μg of total RNA was used as starting material, using the Bio-Rad iScript cDNA Synthesis Kit. The reaction mix contained 4 μl 5× iScript Reaction mix, RNA, and 1 U of Reverse Transcriptase in 20 μl reaction volume following the manufacturer’s temperature profile. Quantitative PCR reactions were carried out in 20 μl volumes in a 96-well plate (Applied Biosystems) containing 1× buffer and other PCR ingredients in SsoAdvanced Universal SYBR Green Supermix from Bio-Rad using an Applied Biosystem qPCR machine. Human GAPDH was used as the internal reference gene, and relative mRNA levels of candidate genes were determined using the 2(-ΔΔCT) method. Three independent experiments were performed. Statistical significance was calculated using unpaired *t* test, *P* < 0.05.

### Library preparation, preprocessing, and alignment for RNA-sequencing

Purified total RNA was used to prepare Libraries following the protocol using Qiaseq Stranded RNA lib Kit with UDI and QIAseq FastSelect -rRNA HMR Kit (Qiagen INC.) for Illumina sequencing. Libraries were QC using Fluorometric Quantitation (Qubit; Invitrogen:ThermoFisher Scientific), Agilent 2100 bioanalyzer (Agilent) and QPCR (Roche Light Cycler). RNA-seq libraries were multiplexed and sequenced as 2 ×75 bp paired-end on NEXTSEQ 500 (Illumina) following standard protocols. Sequencing data was QC and analysed using GSNAP (http://research-pub.gene.com/gmap/) software for mapping to a reference genome and HTSeq-count (http://www-huber.embl.de/users/anders/HTSeq/) to assign unique counts to ENSEMBL annotated transcripts and Bioconductor packages*DESeq* (http://bioconductor.org) to determine the relative counts of sequences from each gene relative to each other (transcriptional profiling), normalisation and statistical comparison etc. RNA sequencing data were downloaded from BGI. Adaptor sequences were first trimmed from the RNA sequencing reads using Trimgalore v0.3.7 and sequence quality was assessed using fastqc v.0.11.4. Trimmed reads were then aligned against human genome hg38 using STAR v2.5.1. Gene counts were also calculated with STAR. Pairwise differential analysis was performed on raw gene counts to compare gene expression profile between samples with different conditions using R package DESeq2 v1.28. Differential genes were considered significant if the fold change is more than 2.

### Differential expression gene analysis

The statistical analysis for gene expression data was carried out using the R statistical software (version 4.2.0). Differentially expressed genes (DEGs) between bulk, E/M and M were identified using likelihood ratio test for the coefficient. The differential expression gene analysis was carried out using a Wilcoxon rank-sums test with Benjamini–Hochberg test for multiple testing correction. DEGs with an adjusted *P* value less than 0.05 were classified as differentially expressed.

### Principal component analysis

Principal component analysis was performed on the differentially expressed genes between bulk, E/M and M, to reveal a similar degree of transcriptional shift within the three groups.

### Ingenuity pathway analysis

The differentially expressed genes between bulk, E/M and M were uploaded into Ingenuity Pathway Analysis (IPA) software for core analysis. Firstly, to investigate the potential genes that might affect global expression, the upstream regulator analysis was performed by overlaying the differentially expressed genes in Ingenuity Knowledge Database, which allows IPA to determine the activation or inhibition of potential upstream regulators. A Z-score greater than +2 or less than −2 was used as a cut-off to predict activation or inhibition.

### Transcription factor enrichment analysis

The FOXC2 motif was obtained from the JASPAR 2022 database and used to search for genome-wide binding sites against the human hg19 genome using the HOMER (v4.11) module findMotifs. Hits of predicted FOXC2 binding sites near TP63 transcription start sites were found in both JASPAR and HOMER databases. The H3K4Me3 and H3K27Ac histone mark ChIP-seq profiles from ENCODE were also used to indicate the active transcription regulatory regions and active enhancer regions of TP63, respectively.

### ATAC-sequencing

The ATAC-seq protocol was adapted from LaFave et al [23]. Briefly, 50,000 cells were transposed with an all-in-one transposition buffer (Tris pH 7.5, MgCl_2_, DMF 5%, PBS 0.3×, NP-40 0.1%, Illumina Tn5 1×, ddH_2_0 to 50 μL). The transposition reaction was performed at 37 °C for 30 min at 300 rpm on a thermoshaker. Transposed DNA was purified with MinElute column clean-up (Qiagen), then minimally amplified for sequencing by PCR as previously described [24]. Prepared libraries were purified with MinElute column clean-up (Qiagen), digested with ExoI (NEB), and quantified with a KAPA universal library quantification kit (Roche). Libraries were sequenced on the Next-seq platform (Illumina) using a 75-cycle kit. Reads were trimmed and aligned using the NF-Core ATAC-seq pipeline (v2.1.2). TF motif scores were computed for individual samples using chromVAR [25]. Background peaks were sampled (*n* = 250 iterations) to adjust for GC bias and overall accessibility was determined for each peak, which were used to compute motif and k-mer accessibility deviation Z-scores using the computeDeviations function in chromVAR (v1.20.2).

### Statistical analysis

Results represent the mean ± SEM for indicated experiments with at least three independent biological replicas. The statistical methods used are described in the figure legends. Most data were analysed by the unpaired two-tailed Student’s *t* test to compare two groups with significance set at the *P* value < 0.05 unless otherwise stated. Statistical analysis was performed using the Prism 9 (GraphPad) software.

## Results

### The partial EMT state is enriched in TCF1 expression which promotes tumour stemness and metastasis

**Fig. 1:**
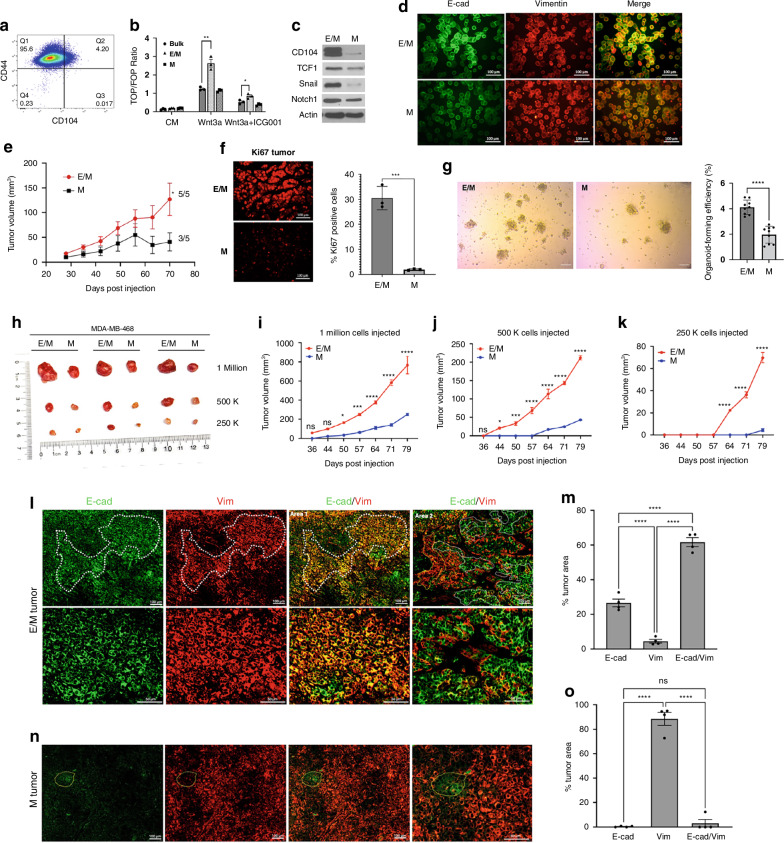
The partial EMT state in MDA-MB-468 cells is enriched in TCF1, which promotes a CSC-like phenotype. **a** CD104 and CD44 FACS profiles of MDA-MB-468. **b** TOP/FOP Flash in MDA-MB-468 bulk (circle), E/M (triangle), and M (square), that were treated with (CM: condition medium), Wnt3a or Wnt3a + ICG001; mean ±  SEM; *P < 0.05; **P < 0.01. **c** Immunoblots (IB) of CD104, TCF1, Snail, Notch1, β-actin in E/M and M cells. **d** MDA-MB-468 E/M cells (top) and M cells (bottom) were co-immunostained for E-cad (FITC) and Vimentin (TRITC) shown in a representative sample out of 3. **e** Mammary tumour growth (volume) post m.f.p injection of E/M or M cells into athymic female nude mice (*N* = 5 each); mean ± SEM; *P* < 0.05. **f** E/M (top) and M (bottom) tumours were immunostained for Ki67; the percent of Ki67 positive cells was calculated in three tumours as mean ± SEM; **P < 0.01. **g** Organoids formed ex vivo by E/M or M tumours; 100 μm; mean ± SEM, ****P < 0.0001. **h** Digital images of mammary tumours formed by E/M and M cell populations (1 × 10⁶, 5 × 10⁵, and 2.5 × 10⁵) sorted from MDA-MB 468. **i**–**k** Tumour growth curves of E/M and M tumours formed by mammary fat pad injection of **i** 1 × 10⁶, **j** 5 × 10⁵, and **k** 2.5 × 10⁵ cells in three mice each. Data are shown as mean tumour volumes ± SEM; ns, *P < 0/05, ***P < 0.001, ****P < 0.0001, as measured by two-way ANOVA. **l**–**o** E/M and M tumours (*N* = 3) were immunostained for E-cad (FITC) and VIM (TRITC). **l** Demarcated area 1 of E/M tumours containing E-cad + /VIM+ clusters vs area 2 with E-cad+ or VIM+ clusters are shown (**l**; upper panels). Corresponding high magnification images of area 1 and area 2, 50 μm, are shown (**l**; bottom panels). **n** M-tumours show mostly VIM staining with minor demarcated areas that are E-cad + , 50 μm. **m**, **o** The percentage of tumour surface area covered by E-cad, E-cad/VIM or VIM expression in E/M tumours (**m**) or M-tumours (**o**) was calculated as mean ± SEM. Data were analysed using one-way ANOVA; ns, no significance, ****P < 0.0001.

TCF1 is aberrantly expressed in a subset of aggressive breast carcinomas and is associated with poor prognosis [26]. TCF1 is encoded by the TCF7 gene, known to act as a critical stemness regulator of T-cell immunity [27]. Cancer cells residing in a partial EMT state were isolated from the basal-like breast cancer subtype based on CD104 (β4 integrin) (ITGB4) and CD44 cell surface expression. CD104^high^ CD44^high^ cells were endowed with tumour regenerative and metastatic potential relative to M cells (CD104^low^ CD44^high^) or E cells (CD104^high^ CD44^low^) [12]. To investigate TCF1 inputs into partial EMT (p-EMT), we FACS-sorted E/M and M cells from the MDA-MB-468 basal-like breast cancer cell line using CD104/CD44 differential surface expression. Interestingly, E/M cells were ~4.2% as compared to M cells which were ~95.6% of the bulk population (Fig. 1a). Of note, E/M cell fraction ranged between 0.8 and 8% likely due to cell plasticity in vitro. E/M cells were enriched in Wnt/β-cat signalling, as shown by a twofold increase in Wnt3a-stimulated TOP/FOP-Flash reporter activation relative to M or bulk cells (Fig. 1b), which was in turn reversed by the Wnt inhibitor ICG001 (Fig. 1b). Interestingly, TCF1 was enriched in E/M cells which overexpressed CD104 as well as Snail and Notch1 (Fig. 1c), which are both p-EMT and stemness markers [12, 28, 29]. Consistent with an epithelial component, E-cad expression was higher in E/M cells than in E/M cells relative to VIM expression which was higher in M than in E/M cells (Fig. 1d). We next tested the tumorigenic and stemness properties of E/M cells relative to M cells. E/M cells produced large mammary tumours in 100% of female athymic nude mice as compared to M cells which formed smaller tumours in 60% of the mice (Fig. 1e), that were less proliferative (Ki67) (Fig. 1f). In support of stemness, E/M tumours formed robust organoids ex vivo as compared to M-tumours (Fig. 1g). Moreover, E/M cells formed tumour-initiating potential and formed large mammary tumours relative to M cells, at any of the injected limiting cell dilutions (1.0, 0.5, 0.25 × 10^6^) (Fig. 1h–k). Of note, E/M and M cells were injected into the contralateral mammary glands of female NSG (NOD scid gamma) mice to avoid random variation in tumour cell intake between individual mice. Hence, these data support the notion that E/M cells are endowed with stem and self-renewal capability, underlying organoid formation and tumour-initiating potential, relative to M cells which were locked in a terminally differentiated benign state.

Interestingly, the partial EMT phenotype was relatively stable in vivo as shown by the abundance of CD104/CD44/E-cad/VIM positive areas in E/M tumours (Fig. S1A). By comparison, M-tumours contained mostly areas that were CD104^low^ CD44^high^ lacking E-cad/VIM with minor CD104/CD44/E-cad/VIM positive clusters (Fig. S1B**)**. In support of these data, staining for E-cad/VIM showed that the bulk of the E/M tumour was E-cad/VIM positive (61.6%) relative to E-cad (26.5%) or VIM (4.5%) positive (Fig. 1l, m), while the remaining of the tumour was unstained due to necrosis. By contrast, M-tumours were abundantly VIM+ (92%) relative to E-cad+ (1.36%) or E-cad + /VIM+ (2.80%) areas (Fig. 1n,o). By comparison, in vitro culturing of MDA-MB-468 E/M cells (7.3%) or M cells (50%) (Fig. S1C) for 15 days, resulted in E/M cells shifting into M-state (51.3%) while keeping a constant pool of E/M cells (6.53%) (Fig. S1E). By contrast, M cells gave rise mostly to M cells (73%) and very low levels of E/M cells (1.5%) (Fig. S1D). These data support the notion that E/M cells are capable of self-renewing and differentiating into E-state in vivo and M-state in vitro. These data implied that E/M to M differentiation might be a default state in vitro, due to lack of the tumour microenvironment that supports epithelial differentiation in vivo. Lastly, E/M cells gave rise to lung metastasis at ~4 months post-surgical excision of the primary tumour (at 1 cm diameter), which prevents mouse morbidity by excess tumour burden. Namely, E/M tumours produced lung mets in 100% as compared to M cells in 33% of the mice (Fig. S1F). Thus, these findings underscored the notion that E/M cells act as cancer stem-like cells which are endowed with tumorigenic, stem and metastatic abilities.

To test the effect of TCF1 on hybrid EMT, we overexpressed (OE) TCF1 in MDA-MB-468 cells (Fig. S2A). Remarkably, TCF1OE cells showed striking upregulation of CD104 and CD44 (Fig. S2A) as well as increased Wnt3a/TOP/FOP activation (Fig. S2B). Consistent with these data, TCF1OE cells exhibited a 12-fold increase in CD104^high^CD44^high^ cells relative to control cells (Fig. S2C). In vivo, TCF1OE cells gave rise to substantial mammary tumours in 100% of the mice as compared to control MDA-MB-468 cells which formed small tumours in 60% of the mice (Fig. S2D). Moreover, TCF1OE cells produced lung mets following primary tumour resection in 83% of the mice as compared to 20% by control cells (Fig. S2E). Consistently, TCF1OE tumours formed robust organoids ex vivo (Fig. S2F) and co-expressed CD104/CD44 with E-cad/VIM as compared to control tumours (Fig. S2G). These data highlight the dramatic effect of TCF1 on partial EMT induction, likely due to potentiation of canonical Wnt signalling driving stemness and metastasis. Furthermore, TCF1 OE in the normal MCF10A mammary cell line increased by 5-fold Wnt3a/TOP/FOP signalling (Fig. S3A, B), and caused striking increases in epithelial (E-cad) and mesenchymal (N-cad, Snail, Nanog) markers (Fig. S3A). Of note, MCF10A/TCF1OE cells were chemotactic towards EGF, consistent with EGFR/pEGFR upregulation in these cells (Fig. S3C, D). Moreover, TCF1 OE cells formed robust mammospheres (Fig. S3E), which was consistent with a 2-fold increase in the classical CD44^high^/CD24^low^ mammary stem cell population (Fig. S3F). Thus, TCF1 was able to elicit a partial EMT phenotype in cancer and normal mammary cells.

### RNA sequencing unravels FOXC2 as one of the most upregulated transcription factors in E/M cells

**Fig. 2:**
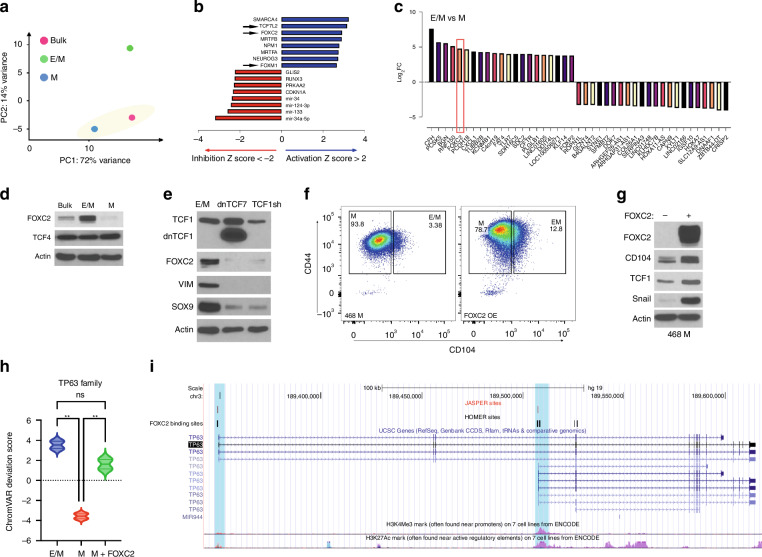
RNA-seq unravels FOXC2 as an EMT transcription factor that is restricted to E/M cells. **a** Principal Component Analysis of MDA-MB-468 bulk (pink), E/M (green) and M (blue) cells. **b** Upstream regulator analysis by IPA comparing RNA-seq datasets between E/M and M cells. Arrows indicate the top-upregulated genes TCF7L2 (TCF4), FOXC2, and FOXM1 in E/M cells. **c** Differential mRNA expression analysis comparing E/M and M cells, highlighting FOXC2 (orange). **d** Immunoblots of FOXC2 and TCF4 in bulk, E/M, and M cells. **e** IB of TCF1, dnTCF1, FOXC2, VIM, and SOX9 in E/M cells expressing empty vector, dnTCF7 or TCF1sh. **f** CD104 and CD44 FACS profiles of M cells and FOXC2-OE M cells. **g** IB of FOXC2, CD104, TCF1, Snail in M cells vs FOXC2-OE M cells. **h** Violin plot of chromatin accessibility of *Trp63* motif family (TP53, TP73) comparing ATAC-seq datasets from MDA-MB-468 E/M, M, and FOXC2-OE M cells (**P < 0.01). **i** Predicted FOXC2 binding sites (blue) are located near the transcription start sites (TSS) of the two major TP63 isoforms. Red line: predicted FOXC2-binding sites found in JASPAR TF Binding Site database. Black line: predicted FOXC2 binding sites found in HOMER module findMotifs database. H3K4Me3: peaks of trimethylation to lysine 4 on histone 3 from ENCODE. H3K27Ac: peaks of acetylation of the lysine residue at N-terminal position 27 of histone 3 from ENCODE.

To elucidate novel transcriptional regulators of the partial EMT state, we performed RNA sequencing comparing MDA-MB-468 E/M and M subpopulations sorted by CD104/CD44 expression from bulk cells. Principal component analysis (PCA) revealed divergent transcriptomics between E/M and M or bulk cells (Fig. 2a). To unravel the putative upstream regulators of the hybrid E/M state, we analysed the differentially expressed genes (DEGs) between E/M and M cells in Ingenuity Pathway Analysis software. Interestingly, TCF7L2 (TCF4), FOXC2 and FOXM1 ranked among the top upregulated genes in E/M vs M cells (Z-score >2) (Fig. 2b). Interestingly, SMARCA4, an ATPase subunit of the SWI/SNF chromatin remodelling complex, was upregulated in E/M cells, pointing to a potential epigenetic regulation of p-EMT via TCF7 [30] (Fig. 2b). These data implied that Wnt signalling (TCF4), EMT (FOXC2) and proliferation (FOXM1) act in concert to orchestrate the p-EMT state. By contrast, Mir-34 and Mir-34a-5p, two negative regulators of Snail, were downregulated in E/M cells (Z-score <2) (Fig. 2b), supporting the paradigm that microRNAs feed into a network regulating the p-EMT state by Snail [31]. Importantly, RNA-seq datasets pointed to FOXC2 mRNA and protein amongst the top five upregulated genes in E/M cells relative to unchanged TCF4 (Fig. 2c, d). Moreover, inhibition of TCF1 by shRNA or dominant-negative (dn) TCF7, which lacks the β-catenin binding domain, in E/M cells suppressed FOXC2 expression as well as VIM and SOX9 (Fig. 2e), two Wnt target genes [32], thus underscoring a link between FOXC2 and TCF1/Wnt signalling in regulating hybrid EMT.

### FOXC2 promotes M to E/M differentiation via p63 isoform upregulation

The partial EMT state may be inherently plastic, allowing E/M cells to shift between E and M state in response to contextual TME signals. To test whether FOXC2 induces M to E/M transition we overexpressed (OE) FOXC2 in M cells from MDA-MB-468. This increased the percentage of the E/M (CD104^high^CD44^high^) cell fraction by 3.5-fold (Fig. 2f), consistent with upregulation of CD104, TCF1 and Snail (Fig. 2g), implying FOXC2 promotes M to E/M transition.

Next, we sought to elucidate potential candidate genes downstream of FOXC2, which may contribute to the partial EMT state, especially in the context of canonical Wnt signalling. This led us to investigate whether *Trp63*, a bona fide Wnt target gene and a pivotal regulator of the E/M state [33], was activated by FOXC2. This idea was initially supported by ATAC-seq demonstrating increased chromatin accessibility at p63 and p73 motifs in E/M relative to M cells (Fig. 2h), and in M cells overexpressing FOXC2 relative to M control cells (Fig. 2h). Moreover, global genome mining of genes with putative FOXC2 binding sites revealed that the promoter and enhancer regions of TA and ΔN p63 isoforms contained FOXC2 binding motifs (Fig. 2i). These findings implied that FOXC2 regulates p63 gene transcription.

**Fig. 3:**
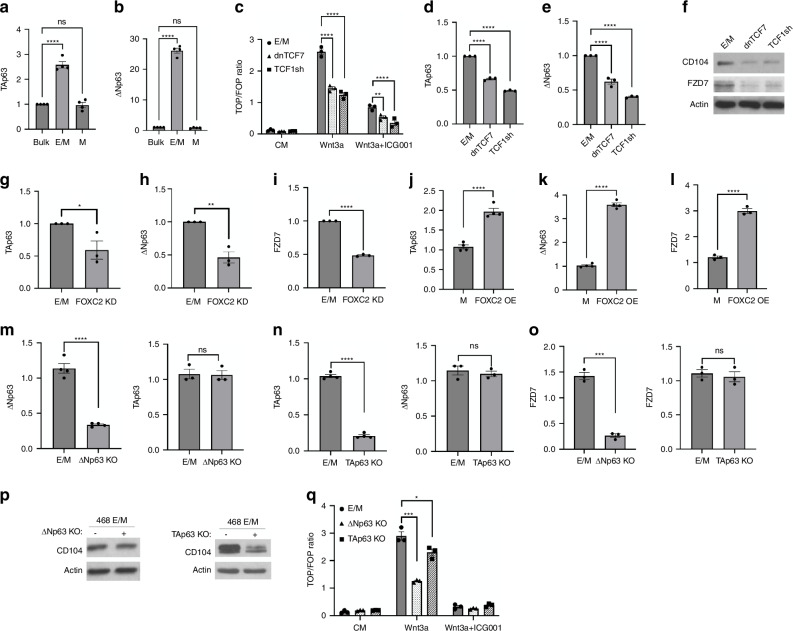
FOXC2 promotes p63 isoform expression in hybrid EMT cells. **a**, **b** TAp63 and ΔNp63 mRNA fold change in MDA-MB-468 bulk, E/M, and M cells; mean ± SEM (ns, ****P < 0.0001). **c** TOP/FOP Flash in E/M control cells (circle) vs E/M cells with dnTCF7 (triangle), or TCF1sh (square), that were untreated (CM) or treated with Wnt3a or Wnt3a + ICG001; mean ± SEM; **P < 0.01; ****P < 0.0001. **d**, **e** TAp63 and ΔNp63 mRNA fold change in dnTCF7 or TCF1sh E/M cells relative to control E/M cells; mean ± SEM; ****P < 0.0001. **f** IB of CD104 or Frizzled 7 in E/M control cells, dnTCF7 or TCF1sh E/M cells. **g**–**i** TAp63, ΔNp63, Frizzled 7 mRNA fold change in E/M-FOXC2KD cells relative to control E/M cells; mean ± SEM; *P < 0.05, **P < 0.01, ****P < 0.0001. **j**–**l** TAp63, ΔNp63, and Frizzled 7 mRNA fold change in M control and FOXC2-OE M cells; mean ± SEM; ****P < 0.0001. **m** ΔNp63 or TAp63 mRNA fold change in ΔNp63KO E/M cells vs control E/M cells; mean ± SEM; ****P < 0.0001; ns. **n** TAp63 or ΔNp63 mRNA fold change in TAp63KO E/M vs control E/M cells; mean ± SEM; ****P < 0.0001; ns. **o** Frizzled 7 mRNA fold change in ΔNp63KO or TAp63KO vs control E/M cells; mean ± SEM, ****P < 0.0001; ns. **p** IB of CD104 in E/M cells expressing ΔNp63 KO or TAp63 KO. **q** TOP/FOP Flash in E/M control cells (circle), ΔNp63KO (triangle), and TAp63KO (square) cells, untreated (CM) or treated with Wnt3a or Wnt3a + ICG001; mean ± SEM, *P < 0.05; ***P < 0.001. Unpaired *t* test was used to compare two groups; one-way ANOVA, Dunnett’s was used for multiple comparisons test. ns no significance.

Next, we sought to analyse the relative distribution of the p63 isoforms in E/M vs M cells and the relationship between FOXC2 and p63 isoforms. Due to a lack of non-discriminatory isoform-specific p63 antibodies to detect protein, we measured mRNA expression. Like FOXC2, TAp63 and ΔNp63 were both upregulated in E/M cells relative to M or bulk MDA-MB-468 cells (Fig. 3a, b). Of note, ΔNp63 mRNA was increased by tenfold as compared to TAp63 mRNA in E/M cells, thus consistent with tumour-promoting vs suppressive functions of ΔNp63 vs TAp63 [34–36]. Moreover, inhibition of Wnt signalling by TCF1shRNA or dnTCF7 in E/M cells (Fig. 3c) reduced TAp63 and ΔNp63 mRNA (Fig. 3d, e), and in turn CD104 and Frizzled 7 protein (Fig. 3f), thus supporting that TCF1/Wnt signalling regulates p63 isoform expression in E/M cells, thereby promoting epithelial stemness [37]. Further, to determine whether FOXC2 regulates p63 isoforms, we knocked down FOXC2 by siRNA in E/M cells and overexpressed FOXC2 in M cells. FOXC2 KD in E/M cells reduced TAp63 and ΔNp63 (Fig. 3g, h) as well as Frizzled 7 mRNA (Fig. 3i). By contrast, FOXC2 OE in M cells reversed these effects (Fig. 3j–l), thus underscoring the regulation of p63 isoforms by FOXC2.

Next, to assess whether p63 isoforms regulate the E/M state, we performed CRISPR-Cas9 isoform-specific knockout in E/M cells (Fig. 3m, n). Of note, ΔNp63 KO did not affect the expression of TAp63 and vice versa, which rules out functional redundancy between the two p63 isoforms (Fig. 3m, n). In fact, ΔNp63 KO suppressed Frizzled 7 mRNA but had no effect on CD104 protein, whereas TAp63 KO suppressed CD104 protein but had no effect on Frizzled 7 mRNA (Fig. 3o, p). Consistent with these data, ΔNp63 KO strongly reduced Wnt3a/TOP/FOP activation as compared to mild attenuation by TAp63 KO (Fig. 3q). These data underscored that FOXC2 regulates TAp63 and ΔNp63, which activate the epithelial programme and Wnt signalling via upregulation of CD104 and Frizzled 7, respectively, two key determinants of the hybrid EMT state [11, 35].

### Wnt3a promotes transient ERK phosphorylation, resulting in FOXC2 and FOXM1 upregulation

**Fig. 4:**
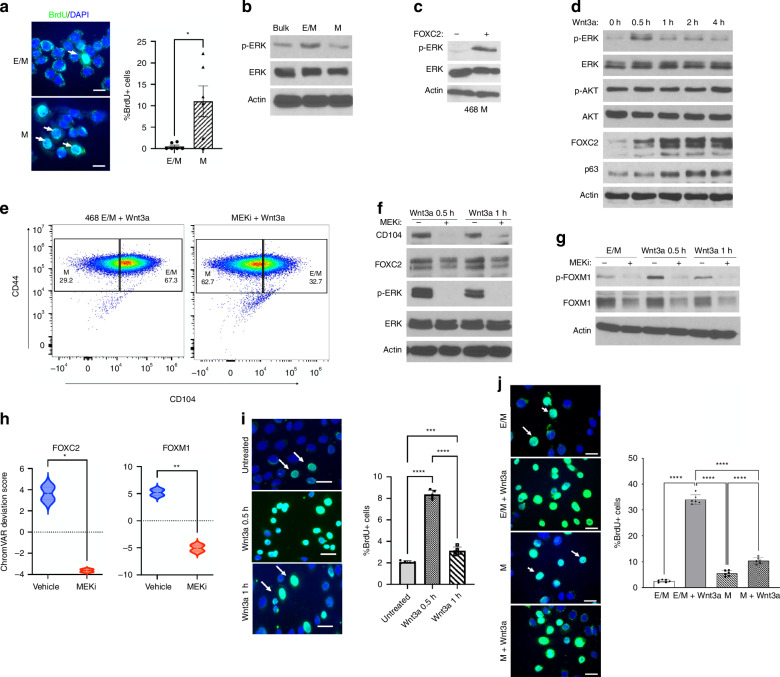
Wnt3a promotes transient p-ERK to promote E/M state maintenance and self-renewal via FOXC2 and FOXM1, respectively. **a** BrdU staining of MDA-MB-468 E/M and M cells grown in 10% FBS/RPMI. BrdU (green), DAPI (blue); 20 μm. The percent of BrdU-positive cells (white arrows) is shown as mean ± SEM, unpaired *t* test; *P < 0.05. **b** IB of p-ERK, ERK in bulk, E/M, and M cells. **c** IB of p-ERK, ERK in M cells and FOXC2-OE M cells. **d** IB of p-ERK, ERK, p-AKT, AKT, FOXC2, p63 in E/M cell stimulated with 100 ng/ml Wnt3a in 0.2% FBS/RPMI at the indicated time points. **e** CD104 and CD44 FACS profiles of E/M cells treated with vehicle (DMSO) or 1 μM MEK1 inhibitor (MEKi) PD0325901 (PD901) for 24 h, followed by 100 ng/ml Wnt3a in 0.2% FBS/RPMI for 0.5 h. **f** IB of CD104, FOXC2, p-ERK, ERK in E/M cells treated with DMSO or 1 μM of PD901 for 24 h, and stimulated with 100 ng/ml Wnt3a for 0.5 h or 1 h. **g** IB of p-FOXM1 (Ser35), FOXM1 in E/M cells treated as in (**f**). **h** Violin plots of chromatin accessibility of FOXC2 or FOXM1 motifs in MDA-MB-468 E/M cells treated with DMSO or 1 μM PD901 for 24 h; *P < 0.05; **P < 0.01. **i** BrdU staining of E/M cells treated without or with 100 ng/ml Wnt3a in 0.2% FBS/RPMI for 0.5 h or 1 h; BrdU (green); 20 μm. The percent of BrdU-positive cells (arrows) is shown as mean ± SEM, one-way ANOVA, Tukey’s multiple comparisons test, ***P < 0.01, ****P < 0.001. **j** BrdU staining of E/M cells (top two panels) and M cells (bottom two panels) that were treated with 5 ng/ml Wnt3a for 0.5 h; the percent of BrdU-positive cells (arrows) is shown as mean ± SEM, one-way ANOVA, Tukey’s multiple comparisons test, ****P < 0.001.

Beyond using the FOXC2/p63 axis to maintain the partial EMT state, Wnt signalling promotes self-renewal. Clearly, E/M cells were less mitotic than M cells as shown by the lower level of BrdU+ cells, consistent with a slow cycling CSC-like state (Fig. 4a). In search of signalling pathways governing the proliferation of E/M cells, we found that basal MAPK/ERK phosphorylation (p-ERK) was higher in E/M than in M cells, relative to unchanged ERK levels (Fig. 4b). Moreover, FOXC2 OE in M cells upregulates p-ERK, likely supporting M to E/M transition (Fig. 4c). In line with ERK activation, Wnt3a stimulated transient ERK phosphorylation in E/M cells, which peaked by 0.5 h and declined to basal level by 1 h post Wnt3a addition, while having no effect on Akt phosphorylation (Fig. 4d). These data underscored a role for temporal Wnt/ERK activation in E/M programming, which was further supported by sustained FOXC2 and p63 upregulation in E/M cells (Fig. 4d). In fact, MEK/ERK inhibition by PD 0325901 (PD901) in E/M cells diminished the fraction of CD104^high^CD44^high^ cells by 50%, whether untreated (not shown) or treated with Wnt3a for 0.5 h (Fig. 4e). This led to a proportional increase in the M-cell pool (CD104^low^ CD44^high^) (Fig. 4e), coinciding with reduced CD104 expression (Fig. 4f). However, MEK1 inhibition by PD901, only mildly reduced FOXC2 expression by 0.5–1 h of Wnt3a treatment, implying ERK does not critically regulate FOXC2 expression (Fig. 4f). By contrast, phosphorylation and expression of FOXM1, a TF known to be pivotal for cell proliferation [21, 22], was inhibited by PD901 at 0.5 h and 1 h post Wnt3a treatment (Fig. 4g), indicative of activation and stabilisation of FOXM1 by p-ERK in E/M cells [19, 20]. Interestingly, however, ATAC-seq of E/M cells revealed open chromatin accessibility sites at the FOXC2 and FOXM1 motifs that were interestingly shut-down by PD901 (Fig. 4h), implying phosphorylation by ERK regulates accessibility to these regions. Curiously, while PD901 suppressed chromatin accessibility at the FOXC2 motif, it had a negligible effect on FOXC2 expression, implying p-ERK regulates FOXC2 gene activity and not protein stability.

In support of the notion that transient p-ERK promotes proliferation, treatment of E/M cells with Wnt3a stimulated S-phase entry, as measured by BrdU uptake, which peaked by 0.5 h and declined to low levels by 1 h post Wnt3a treatment (Fig. 4i). By contrast, treatment of M cells with Wnt3a had a negligible effect on M-cell proliferation (Fig. 4j), thus underscoring the low sensitivity of M cells to Wnt3a relative to E/M cells. These results highlight an intricate regulation of partial EMT by Wnt3a, in part, due to transient p-ERK, which stimulates FOXC2 and FOXM1 gene activity to drive stemness and self-renewal, respectively.

### Wnt3a stabilises FOXC2 via Wnt/CDK4/6 mediated activation of mammary tumour growth

**Fig. 5:**
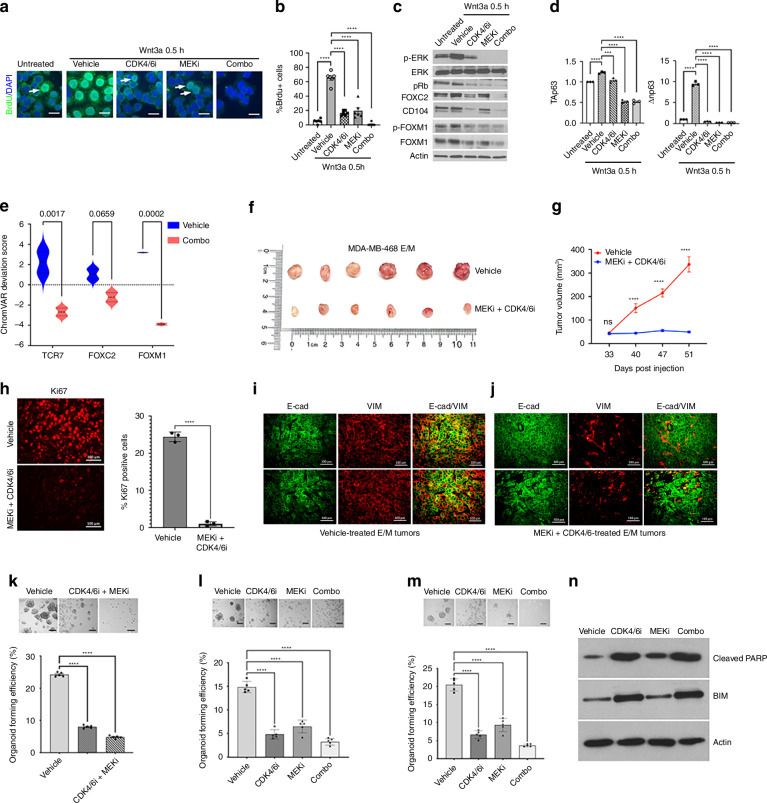
CDK4/6/ERK inhibition suppresses E/M stemness and self-renewal, via FOXC2 and FOXM1 inhibition, resulting in epithelial differentiation and suppression of mammary tumour growth. **a** BrdU-stained E/M cells treated with DMSO, 10 μM palbociclib (CDK4/6i), 1 μM PD901 (MEKi), or combination for 24 h, followed by 100 ng/ml Wnt3a in 0.2% FBS/RPMI for 0.5 h. BrdU (green), DAPI (blue), 20 μm. **b** The percent of BrdU+ cells (mean ± SEM); one-way ANOVA, Dunnett’s multiple comparisons test (****P < 0.0001). **c** IB of p-ERK, ERK, p-Rb (Ser780), FOXC2, CD104, p-FOXM1 (Ser35), FOXM1 in E/M cells treated with vehicle, 10 μM palbociclib, 1 μM PD901, or combination for 24 h, followed by 100 ng/ml Wnt3a in 0.2% FBS/RPMI for 0.5 h. **d** TAp63 and ΔNp63 mRNA fold change in E/M cells that were treated with vehicle, 10 μM palbociclib, 1 μM PD901, or combination for 24 h, followed by 100 ng/ml Wnt3a 0.2% FBS/RPMI for 0.5 h; mean ± SEM; One-way ANOVA, Dunnett’s multiple comparisons test; ***P < 0.001; ****P < 0.0001. **e** Violin plot of chromatin accessibility of TCF7, FOXC2, and FOXM1 motifs in MDA-MB-468 E/M cells that were treated with vehicle or 10 μM palbociclib+1 μM PD901 (combo) for 24 h, followed by 100 ng/mL Wnt3a in 1% FBS/RPMI for 0.5 h. The calculated *P* value using the two-way ANOVA test is shown on the panel. **f** Digital images of tumours resected from mice following m.f.p injection of MDA-MB 468 E/M cells. The upper lane shows the vehicle control group and the lower lane displays tumours from mice treated with MEKi+CDK4/6i (20 mg/kg PD901 + 50 mg/kg Abemaciclib). **g** Tumour volumes were measured by comparing the vehicle control and drug-treated groups. Data are shown as mean ± SEM; *P* value was determined by the two-way ANOVA *t* test (ns, ****P < 0.0001). **h** Vehicle vs (MEKi+CDK4/6i)-treated mammary tumours (*n* = 3 each) were immunostained for Ki67; the percent of Ki67+ cells is shown as mean ± SEM; *P* < 0.05. **i**, **j** Vehicle (**i**) vs MEKi+CDK4/6i (**j**) treated E/M tumours (*N* = 3 each) were immunostained for E-cad (FITC) and Vimentin (TRITC). Two representative images are shown for each condition. **k** Ex vivo organoids from E/M tumours that were treated with vehicle (left), or 10 μM Abemaciclib + 1 μM PD901 (combo) (middle), or re-treated in vitro with combo (right) for 5 days in organoid growth media;100 μm; mean ± SEM, one-way ANOVA, Dunnett’s multiple comparisons test; ****P < 0.0001. **l** Organoid cultures from vehicle-treated tumours were untreated (left), treated as indicated at seeding in vitro for 5 days to grow (**m**) or 3 days post seeding for 7 days in organoid growth media; 100 μm; mean ± SEM, one-way ANOVA, Dunnett’s multiple comparisons test; ****P < 0.0001. **n** Organoid cultures from (**l**) were analysed by immunoblotting for Cleaved-PARP and Bim expression.

In light of the differential effects of Wnt/ERK on FOXM1 *vs* FOXC2 activation in E/M cells, we searched for candidate kinases that might tightly regulate FOXC2. As FOXC2 and FOXM1 contain ERK and/or CDK4/6 phosphorylation sites [19, 38], we surmised that transient p-ERK by Wnt3a in E/M cells promotes S-phase via CDK4/6 activation, likely due to Cyclin D1 upregulation [39, 40]. In support of this idea, drug perturbation of MEK/ERK by PD901 or CDK4/6 by palbociclib, markedly attenuated BrdU uptake in E/M cells which was further exacerbated by PD901+palbociclib (combo) (Fig. 5a, b). Consistent with S-phase inhibition, Rb phosphorylation (pRb) was attenuated by PD901 or palbociclib, and especially by drug combination (Fig. 5c). These results were compatible with the notion that p-ERK activates CDK4/6, resulting in pRb, causing G1/S transition [40, 41]. Remarkably, FOXC2 and CD104 expression, and to a lesser extent, also FOXM1 was strongly attenuated by palbociclib+PD901 as compared to either drug alone (Fig. 5c). In line with the FOXC2/p63 axis, ΔNp63 mRNA was dramatically inhibited by PD901 and/or palbociclib relative to TAp63 mRNA which was mostly attenuated by PD901 (Fig. 5d). In fact, ATAC-seq showed that combo-treated E/M cells exhibited a closed chromatin state at TCF7 (*P* = 0.017) and FOXM1 (*P* = 0.0002) motifs, and to a lesser extent, also at the FOXC2 motif (*P* = 0.0659) (Fig. 5e), thus underscoring p-EMT regulation by these genes.

Of note, although MDA-MB-468 cells are RB1 null [42], they expressed the RB-like family members, RBL1 and RBL2 [43], which likely sensitise these cells to CDK4/6 inhibition by palbociclib. This was indicated by RNA-seq (Fig. S4A) and immunoblotting showing expression of RBL2, but not RBL1, in E/M and M cells, as well as pRB in E/M cells (Fig. S4B). Collectively, these results suggested that transient ERK/CDK4/6 activation in E/M cells by Wnt3a results in RBL2 phosphorylation, causing S-phase transition. In parallel, ERK/CDK4/6 upregulates FOXC2 and FOXM1 to promote E/M maintenance and self-renewal, respectively.

### ERK/CDK4/6 inhibition suppresses tumour growth and organoid formation via epithelial differentiation

We next sought to test the therapeutic potential of ERK/CDK4/6 drug inhibition on mammary tumour growth by E/M cells. We used a combination of PD901 and Abemaciclib, a highly potent CDK4/6 inhibitor that was shown to be efficient in palbociclib-resistant ER+ breast tumours [44]. NSG female mice bearing MDA-MB-468 E/M mammary tumours of 0.2 cm diameter were treated daily with vehicle or 20 mg/kg PD901 plus 50 mg/kg Abemaciclib by oral gavage for 3 weeks, 5 days per week, with a 2-day treatment interval between each week. Remarkably, mice that were treated with PD901/Abemaciclib exhibited a dramatic reduction in mammary tumour growth relative to vehicle-treated mice (Fig. 5f, g), which was accompanied with reduced proliferation (Ki67) (Fig. 5h). Interestingly, staining for E-cad/VIM showed that PD901/Abemaciclib-treated tumours shifted from an E-cad^high^/VIM^high^ to E-cad^high^/VIM^low^ state (Fig. 5i, j), indicative of E/M to epithelial (E) differentiation.

Indeed, Abemaciclib/PD901-treated E/M tumours were unable to form organoids ex vivo as untreated (Fig. 5k, 2nd panel) or drug-replenished (Fig. 5k, 3rd panel) in vitro as compared to vehicle-treated E/M tumours. Moreover, treatment of vehicle-treated E/M organoid cultures ex vivo, at the time of seeding, with individual drugs showed that Abemaciclib (CDK4/6i) or PD901 (MEKi), as well as combo regimen were able of suppressing organoids to a similar degree (Fig. 5l). Of note, addition of any of the drugs, 3 days post organoid maturation, was able of disrupting organoids (Fig. 5m). Inhibition of organoids at the time of seeding (Fig. 5l) was associated with increased cleaved-PARP levels especially by CDK4/6i (Abemaciblib), matching increases in pro-apoptotic Bim protein (Fig. 5n), known to be destabilised by CDK4/6 or ERK phosphorylation [45, 46].

### Wnt3a and EGF promote self-renewal vs differentiation via transient vs sustained ERK activation involving EGFR deactivation

**Fig. 6:**
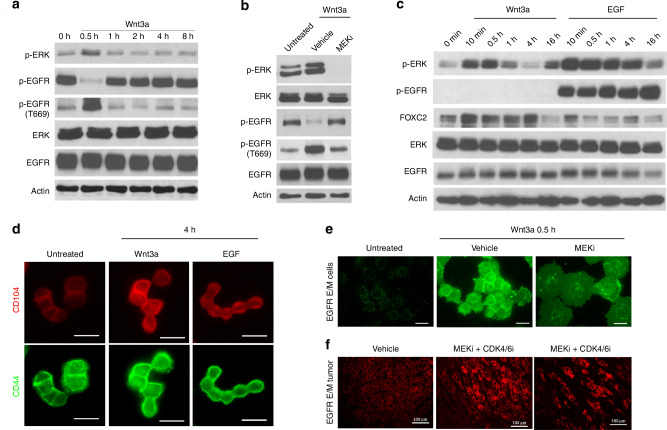
Wnt3a promotes transient p-ERK via EGFR deactivation while EGF promotes sustained p-ERK in E/M cells. **a** IB of p-ERK, p-EGFR (Y1173), p-EGFR (T669), ERK, EGFR in E/M cells stimulated with 100 ng/ml Wnt3a/1%FBS/RPMI at the indicated time points. **b** IB of p-ERK, ERK, p-EGFR (Y1173), p-EGFR (T669), EGFR, in E/M cells treated with vehicle or 1 μM PD901 for 24 h, followed by 100 ng/ml Wnt3a in 1%FBS/RPMI for 0.5 h. **c** IB of p-ERK, p-EGFR (Y1173), FOXC2, ERK, EGFR in E/M cells stimulated with 100 ng/ml Wnt3a or EGF in 0.2%FBS/RPMI at indicated time points. **d** IF for CD104 (red) and CD44 (green) in E/M cells stimulated with 100 ng/ml Wnt3a or EGF for 4 h in 0.2%FBS/RPMI; 20 μm. **e** IF for EGFR (FITC) in E/M cells (top panels) treated with vehicle or 1 μM PD901 (MEKi) for 24 h, followed by 100 ng/ml Wnt3a in 0.2%FBS/RPMI for 0.5 h; 20 μm. **f** EGFR immunostaining (TRITC) in E/M tumours that were treated with vehicle (left panel) as compared to Abemaciclib (CDK4/6i) plus PD901 (MEKi) in vivo (middle and right panel).

Overexpression of EGFR in the MDA-MB-468 cell line [47], including the E/M subpopulation, clearly supports a scenario where EGF is dominant over Wnt3a in promoting ERK signalling, thus questioning how Wnt3a can transiently activate ERK in the presence of EGFR. Interestingly, stimulation of p-ERK by Wnt3a (0.5 h) in E/M cells growing in media/1% FBS, which supports basal EGFR phosphorylation, caused striking inhibition of EGFR tyrosine phosphorylation (pEGFR) (Fig. 6a), which was recovered at 1 h, lasting for 8 h post Wnt3a, matching decreases in ERK phosphorylation (p-ERK) (Fig. 6a). In support of a role for p-ERK in this process, EGFR inhibition by Wnt3a was reversed by PD901 (Fig. 6b). Remarkably, transient inactivation of EGFR tyrosine phosphorylation by Wnt3a (0.5 h) was accompanied by striking EGFR phosphorylation on Threonine 669 (Thr669) (Fig. 6a), which declined upon p-ERK attenuation at 1–8 h post Wnt3a (Fig. 6a), and was in turn suppressed by PD901 (Fig. 6b). This result was consistent with Thr669 as a putative ERK phosphorylation site on the EGFR transmembrane domain which attenuates EGFR tyrosine kinase activation [48]. Thus, our results suggest that transient ERK phosphorylation by Wnt3a in E/M cells inactivates EGFR due to Thr669 phosphorylation, which in turn prevents sustained phosphorylation of ERK by EGF.

Other than self-renewing, CSCs (E/M cells) are programmed to differentiate into progenitors that make up the bulk of the tumour [49]. We propose that Wnt3a/ERK inactivates EGFR, which would otherwise sustain ERK phosphorylation that causes E/M to M differentiation. To test this hypothesis, we treated E/M cells with Wnt3a or EGF in media containing low serum (0.2% FBS) to reduce the threshold of EGFR activation by 1% serum, prior to ligand stimulation. Indeed, in contrast to Wnt3a which temporarily activates p-ERK, EGF stimulated robust p-ERK which was sustained for up to 4 h and was accompanied by prolonged EGFR tyrosine phosphorylation for up to 16 h (Fig. 6c). Of note, Wnt3a stimulated sustained FOXC2 expression for up to 4 h relative to poor activation by EGF (Fig. 6c). Thus, Wnt3a sustains FOXC2 expression which maintains an E/M stem-like state, while activating transient p-ERK that promotes self-renewal. This in turn coincides with EGFR deactivation which prevents sustained ERK activation by EGF from causing E/M to M differentiation that causes CSC exhaustion. In support of this idea, E/M cells showed decreased CD104 membrane expression in EGF-treated cells as compared to Wnt3a-treated E/M cells relative to unchanged CD44 surface expression (Fig. 6d). These data were consistent with EGF causing E/M (CD104^high^CD44^high^) to M (CD104^low^CD44^high^) differentiation (Fig. 6d). Finally, it is noteworthy that EGFR deactivation by p-ERK at 0.5 h post Wnt3a stimulation, was accompanied by striking localisation of EGFR at the cell surface, which was in turn negated by PD901, resulting in intra-vesicular EGFR localisation and a flattened cell morphology (Fig. 6e). This result was in fact consistent with the inability of unphosphorylated EGFR to bind c-CBL for endocytic processing [50, 51]. This finding was recapitulated in vivo as the attenuation of E/M tumour growth by PD901/Abemaciclib was associated with a more robust expression of EGFR relative to vehicle-treated tumours (Fig. 6f). Hence, our data demonstrate an interplay between Wnt and EGF in promoting stemness and self-renewal vs differentiation, which supports the notion that transient ERK activation by Wnt3a promotes self-renewal, whereas sustained p-ERK by EGF promotes E/M to M differentiation [52].

### E/M signalling is activated in the HCC1143 basal-like breast cancer cell line via Wnt/EGF signalling

**Fig. 7:**
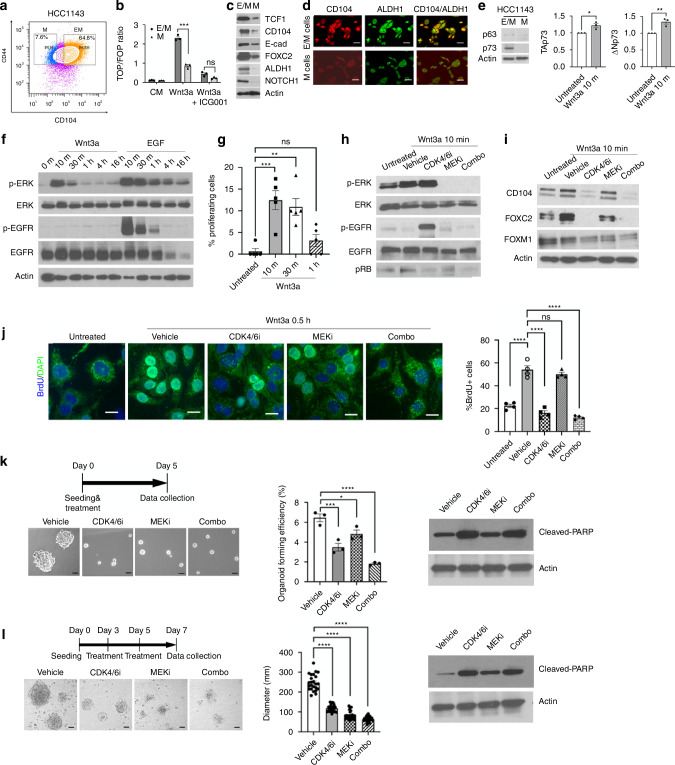
p-EMT signalling in HCC1143 cells via transient ERK/CDK4/6 activation leading to stemness and self-renewal. **a** CD104 and CD44 FACS profiles of HCC1143 cells. **b** TOP/FOP Flash in HCC1143 E/M and M cells, untreated (CM) or treated with Wnt3a or Wnt3a + ICG001; mean ± SEM, unpaired *t* test, ***P < 0.001. **c** IB of TCF1, CD104, E-cad, FOXC2, ALDH1, NOTCH1 in HCC1143 E/M and M cells. **d** E/M and M cells from HCC1143 were co-immunostained for CD104 (TRITC) and ALDH1 (FITC). **e** IB of p63, p73 in E/M and M cells. TAp73 and ΔNp73 mRNA fold change in Wnt3a-treated (10 min) vs untreated E/M cells; *P < 0.05; unpaired *t* test. **f** IB p-ERK, ERK, p-EGFR (Y1173), EGFR in E/M cells stimulated with 100 ng/ml of Wnt3a or EGF in 0.2%FBS/RPMI at the indicated time points. **g** Percent of PCNA + E/M cells untreated or treated with 100 ng/ml Wnt3a in 0.2%FBS/RPMI for 10 min, 30 min, or 1 h; mean ± SEM, one-way ANOVA, Dunnett’s multiple comparisons test; ns; **P < 0.01; ***P < 0.001. **h**, **i** HCC1143 E/M cells were untreated or treated with vehicle, 10 μM palbociclib (CDK4/6i), 1 μM PD901 (MEKi), or combination for 24 h, followed by 100 ng/ml Wnt3a in 0.2% FBS/RPMI for 10 min (except lane1). Cells were IB for **h** p-ERK, ERK, p-EGFR(Y1173), EGFR, p-Rb (Ser780), or **i** CD104, FOXC2, FOXM1. **j** BrdU staining (green) combined with DAPI (blue) of HCC1143 E/M cells that were treated with wnt3a for 0.5 h −/+ indicated drugs; 20 μm. The percent of BrdU+ cells (mean ± SEM); One-way ANOVA, Dunnett’s multiple comparisons test; ****P < 0.0001. **k** Left top: timeline of the drug treatment; HCC1143 cells were seeded and treated with vehicle, 10 μM CDK4/6i, 1 μM MEKi, or combination at Day 0; images were taken at Day 5. Left bottom: organoid images of HCC1143 cells treated as above for 5 days in organoid media; 100 μm. Middle: Organoid-forming efficiency is shown as mean ± SEM; one-way ANOVA, Dunnett’s multiple comparisons test (*P < 0.05; ***P < 0.001; ****P < 0.0001). Right: Cleaved-PARP immunoblots from organoid cultures that were treated with CDK4/6i, MEKi, or both (combo) are shown. **l** Left top: timeline of the drug treatment. HCC1143 cells were seeded at Day 0 and allowed to form organoids for 3 days prior to treatment with the above drugs, which were replenished on Day 5. Left bottom: organoid images on Day 7; 100 μm. Middle: Results are shown as organoid diameter (μm) in each group; mean ± SEM, one-way ANOVA, Dunnett’s multiple comparisons test; ****P < 0.0001. Right: Cleaved-PARP immunoblots from organoids that were treated with CDK4/6i, MEKi, or both (combo) are shown.

We next investigated whether E/M signalling was similarly activated in the HCC1143 basal-like breast cancer cell line. In contrast to MDA-MB-468, which were in average 4% E/M and 75% M cells, HCC1143 cells were 63% E/M (CD104^high^CD44^high^) and 8.3% M-like (CD104^low^CD44^high^) (Fig. 7a) (Fig. S5A). Similarly, HCC1143 E/M cells were enriched in Wnt3a/TOP/FOP reporter activity (Fig. 7b) and TCF1 expression (Fig. 7c) as compared to M cells. Moreover, E/M cells expressed CD104, E-cad, and importantly FOXC2, as well as the ALDH1 and NOTCH1 stemness markers relative to M cells (Fig. 7c). This pattern of gene expression in HCC1143 E/M vs M cells was co-opted by MDA-MB-468 cells, supporting a similarity across distinct TNBC cell lines (Figs. S5B–D and S6A, B). However, these data pointed to a dramatic enrichment in the E/M subpopulation in HCC1143 relative to MDA-MB-468 based on the CD104/CD44 status as well as in the expression level of E/M markers in both cells and organoids (Fig. S6A, B). This led us to investigate E/M heterogeneity across the CD104 spectrum using ALDH1, another marker of epithelial breast cancer stem cells [53–55]. Indeed, ALDH1 expression was high in E/M cells and low in M cells (Fig. 7d) and was interestingly mostly expressed in E/M cells with the highest CD104 expression in HCC1143 cells (Fig. 7d) as well as in MDA-MB-468 cells (Fig. S5E). This finding indicated that the E/M pool might be further stratified by ALDH1 in addition to CD104/CD44. In support of this idea, others used various E/M markers to map a series of hybrid EMT states with differing stem and malignant potential [56]. Interestingly, unlike MDA-MB-468, HCC1143 EM cells were negative for p63 but positive for the related p73 protein [33] (Fig. 7e), known to transactivate CD104 gene expression [57]. Similarly, p73 was expressed as TAp73 and ΔNp73 isoforms in E/M cells, where their levels were increased by Wnt3a (Fig. 7e).

We next investigated whether ERK signalling was differentially activated in E/M vs M cells. Like MDA-MB-468, HCC1143 E/M cells expressed dramatically higher p-ERK levels relative to M cells (Fig. S5F). Next, we tested the differential effects of Wnt3a vs EGF on ERK phosphorylation in E/M cells. Interestingly, Wnt3a induced transient p-ERK which peaked by 10 min and dropped by 30 min to basal levels (Fig. 7f). By contrast, EGF caused robust p-ERK by 10 min, which was attenuated by 1 h, but persisted above threshold levels over 4 h (Fig. 7f), and was associated with transient increases in pEGFR (Fig. 7f). Moreover, Wnt3a caused rapid PCNA/S-phase induction in E/M cells by 10–30 min that waned by 1 h post Wnt3a (Fig. 7g), coinciding with increases in p-ERK and pRB, indicative of CDK4/6 activation (Fig. 7h). Of note, HCC1143 are RB1 null [42], but expressed RBL1, RBL2 and pRB in E/M cells, making them palbociclib-sensitive (Fig. S4C). Interestingly, CDK4/6 inhibition by palbociclib (CDK4/6i) stimulated EGFR tyrosine phosphorylation in HCC1143 E/M cells (Fig. 7h) as well as in MDA-MB-468 E/M cells (Fig. S4D). Consistent with these data, palbociclib suppressed FOXC2 and CD104, and to a lesser extent, also FOXM1 expression (Fig. 7i), thus supporting that CDK4/6i promotes M differentiation. By contrast, PD901 did not increase pEGFR and mildly attenuated FOXC2 or FOXM1 (Fig. 7h, i), implying CDK4/6 is dominant over ERK in inhibiting EGFR in HCC1143. In support of these data, palbociclib suppressed S-phase or BrdU uptake in HCC1143 E/M cells at 30 min post Wnt3a, while PD901 had a negligible effect (Fig. 7j). However, treatment of HCC1143 cells at seeding with palbociclib, PD901, and especially palbociclib+PD901 blocked organoid formation in vitro (Fig. 7k). Furthermore, treatment of mature organoids at 3 days post-seeding, showed that PD901, palbociclib, and palbociclib+PD901 were able of disrupting pre-formed organoids (Fig. 7l). Other than suppressing CD104 and FOXC2 expression (Fig. 7i), treatment of E/M cells with palbociclib+PD901 pre- or post- organoid maturation was associated with increases in cleaved-PARP levels, thereby priming cells for apoptosis (Fig. 7k, l). These results underscored that transient ERK/CDK4/6 activation by Wnt3a upregulates FOXC2 to maintain the hybrid EMT state while activating S-phase and FOXM1 drives self-renewal, supportive of stemness or organoid-forming potential. At last, our data point to a common mechanism used by MDA-MB-468 and HCC1143 basal-like breast cancer cell lines, involving Wnt/ERK/CDK4/6 activation leading to E/M or CSC maintenance and self-renewal combined with inhibition of differentiation. A tentative model summarising our results is included in Fig. S7.

## Discussion

Our cumulative data highlight a pivotal role for TCF1 in orchestrating the partial EMT programme underlying breast cancer stemness and metastasis. TCF1 potentiates canonical Wnt signalling which upregulates FOXC2, and in turn TA and ΔN p63 isoforms, which likely promote epithelial and mesenchymal differentiation via CD104 and Wnt/β-cat signalling activation [35, 58]. Consistent with stemness, E/M cells were endowed with organoid-forming ability, tumour-initiating potential, and metastasis as compared to M cells [9, 10, 12, 19]. We posit that E/M cells act as bona fide cancer stem-like cells that can differentiate into luminal (E) and/or basal-like (M) progenitors in response to cues from the TME. Interestingly, in vitro culturing of E/M or M cells for 15 days showed that E/M cells differentiated into M cells, and not E cells, while maintaining a minor E/M pool. By contrast, M cells produced more M cells and failed to generate E/M or E cells. In vivo, however, E/M cells generated tumours that were predominantly E/M-like (60%) while giving rise to E-like clusters (25%) and M-clusters (5%), relative to M-tumours which were abundantly VIM+ (92%). These data underscored the notion that E/M cells are able of self-renewal and differentiation while M cells are terminally differentiated. We posit that the stability of the hybrid EMT state, might be supported by Snail and Slug upregulation in E/M cells, which repress luminal differentiation in basal-like cells to a degree that still preserves epithelial gene expression, possibly owing to the expression of GRHL2, a “pioneer factor” that increases the transcriptional accessibility of epithelial genes [59]. In addition, FOXC2 may activate NOTCH1 which regulates TCF7 [9, 10], thus enforcing a Wnt signalling loop that supports the p-EMT state.

Functionally, E/M and M cells may fulfil functions that are beyond stemness; with M cells driving cell migration at the leading edge and E/M cells clustering at the core of the tumour [60]. CTCs were interestingly enriched in E/M cells which undergo collective cell migration, thus avoiding anoikis in circulation [61]. M cells may also act as dormant cells which can be re-awakened to differentiate into E/M cells that repopulate the tumour or metastatic sites owing to stemness [62].

TF-mediated regulation of the p-EMT state may be pleiotropic and context-dependent. Intriguingly, the hybrid state was shown to give rise to early and late hybrid states with differential metastatic potential [8, 9]. These studies underscored the complexity of the p-EMT phenotype which spans a spectrum of hybrid EMT states defined by a repertoire of E/M genes. While we used CD104 and CD44 to isolate E/M and M subpopulations, we do not rule out that the CD104^high^CD44^high^ E/M population might be internally heterogenous comprised of various hybrid EMT states. Indeed, using ALDH1, we found that HCC1143 E/M cells expressed ALDH1 at the CD104-highest expressing cells, implying ALDH1 may be one of the markers that can be combined with CD104 to select for a more stringent CSC-like subpopulation. In fact, other studies in skin and mammary tumours have identified EpCAM^-^CD106 ^+^ CD51^+^ cells as a hybrid population enriched in cellular plasticity and aggressiveness [9, 59]. Moreover, studies across various cancers identified N-cadherin, Tenascin-C, P-cadherin, NFATc, NR2F, PDPN and LAMC2 as drivers of the p-EMT state [56]. Thus, further studies are warranted to discriminate universally common from context-dependent markers of hybrid EMT.

We showed that FOXC2 regulates the p-EMT state via p63 isoforms, which was in turn supported by ATAC-seq analysis showing open chromatin accessibility sites at p63/p73 motifs, that were closed in M cells relative to E/M cells and reactivated in M cells overexpressing FOXC2. On the other hand, this analysis revealed that FOXC2 OE in M cells led to a closed chromatin state at the CDx2 locus (Fig. S8A), a tumour suppressor gene frequently deleted in colon CSCs [63]. Moreover, CDx2 was the most significant predictive biomarker of poor survival, thus highlighting the clinical significance of FOXC2 as a CDx2 repressor that might be relevant to basal-like breast cancer [63].

Beyond transcriptionally regulating the E/M state, FOXC2 might recruit TME factors that stabilise p-EMT. Interestingly, p63-positive E/M cells were spotted in head and neck cancers at the leading edge in close contact with CAFs which release ligands (e.g TGFβ) that stimulate EMT on the tumour end [64, 65]. In support of this idea, IPA-based upstream regulator of E/M versus M cells in MDA-MB-468 cells projected that FOXC2 transactivates a plethora of RTKs such as Insulin R, MET, PDGFR and VEGFR in E/M cells, which might be activated by cognate ligands released by the stroma to stabilise the p-EMT state (Fig. S8B).

Importantly, our work evokes a Wnt-regulated programme underlying tumour stemness in hybrid EMT cells via ERK activation, which might be due to RAS stabilisation by Wnt/GSK3β inhibition [66]. Remarkably, the mere activation of Wnt/ERK/CDK4/6 in E/M cells suffices to cement a programme integrating cancer stem cell maintenance (via FOXC2/p63) and self-renewal (via S-phase and FOXM1 activation) with inhibition of differentiation (via EGFR inactivation). Interestingly, the differential effects of Wnt vs EGF on transient vs sustained ERK activation leading to self-renewal vs differentiation are consistent with findings demonstrating that robustness and/or duration of ERK activation determines proliferation vs differentiation outcomes [52]. Of note, EGFR deactivation by ERK results in EGFR recycling to the cell surface, possibly due to the inability of tyrosine-unphosphorylated EGFR to bind c-CBL, which normally promotes EGFR endocytosis [67, 68]. We thus suspect that EGFR TKi-based therapy might be ineffectual or even harmful as it might transiently activate ERK, and in turn fuel tumour stemness and self-renewal.

Of note, FOXM1 and FOXC2 contain ERK and CDK4/6 phosphorylation sites; however, while ERK and CDK4/6 phosphorylate/stabilise FOXM1 [33, 69], the transcriptional regulation of FOXC2 by phosphorylation remains unclear. Although FOXC2 might be transcriptionally activated by p38MAPK or stabilised by PLK1 in CSCs during G2/M transition, we found no activity of these FOXC2 regulatory kinases in E/M cells [70, 71]. However, FOXC2 was shown in lymphatic vascular endothelial cells to harbour eight serine/threonine phosphorylation sites which differentially regulate FOXC2 recruitment to chromatin sites to drive selective gene expression [69]. Our data indicate that FOXC2 might be differentially phosphorylated by ERK and CDK4/6, as shown by greater suppression of FOXC2 expression by palbociclib than PD901. However, chromatin accessibility at the FOXC2 motif was dampened in PD901-treated E/M cells, implying ERK regulates FOXC2 gene activity and not protein stability via phosphorylation. Of note, CDK4/6 inhibition has been approved by the FDA for ER+ breast cancer [72]; however, its effectiveness in TNBC was less clear in light of their negative RB status. Studies in TNBC cell lines have however demonstrated inhibition of tumorsphere formation by palbociclib [73], consistent with the amplification of CDK4/6 or Cyclin D1 in TNBC. In support of these findings, our data show striking attenuation of MDA-MB-468 E/M mammary tumour growth in vivo by PD901/Abemaciclib, which was associated with suppression of organoid formation ex vivo likely due to epithelial (E) differentiation. Interestingly, the success of CDK4/6-based therapy in RB1-negative TNBCs might be due to compensation of RB by RBL proteins, which were indeed expressed in the RB1-deleted MDA-MB-468 and HCC1143 cell lines, implying a diagnostic tool for stratifying TNBC patients for CDK4/6 therapy eligibility. Of note, the scope of CDK4/6-based therapy goes beyond targeting the tumour compartment as it exerts a critical function on the TME by promoting metabolic reprogramming and tumour immunity, which might improve the efficacy of metabolic therapy or immune checkpoint blockade. At last, a limitation of our study is the testing of hybrid EMT cells in immunocompromised mice that does not take into account the interaction of cancer stem cells or CDK4/6-based therapy with the immune microenvironment.

## Supplementary information

Supplemental material

## Data Availability

All materials, reagents or data supporting this manuscript are available upon request from the lead author. RNA and ATAC-seq datasets will be made accessible after revision of the manuscript. The RNA sequencing is available at https://www.ncbi.nlm.nih.gov/geo/query/acc.cgi?acc=GSE274094.
