# Correction: Wnt/ERK/CDK4/6 activation in the partial EMT state coordinates mammary cancer stemness with self-renewal and inhibition of differentiation

**DOI:** 10.1038/s41416-025-03178-z

**Published:** 2025-09-02

**Authors:** Huizhi Liang, Outhiriaradjou Benard, Viney Kumar, Anthony Griffen, Zuen Ren, Kalaiselvi Sivalingam, Jingli Wang, Elena de Simone Benito, Xusheng Zhang, Jinghang Zhang, Kimita Suyama, Lindsay M. LaFave, Larry Norton, Rachel B. Hazan

**Affiliations:** 1 Department of Pathology, Albert Einstein College of Medicine, Bronx, NY USA; 2 Department of Cell Biology, Albert Einstein College of Medicine, Bronx, NY USA; 3 Center for Cancer Research, Massachusetts General Hospital and Harvard Medical School, Boston, MA USA; 4 Francisco de Vitoria University, Madrid, Spain; 5 Computational Genomics Core, Albert Einstein College of Medicine, Bronx, NY USA; 6 Department of Microbiology and Immunology, Albert Einstein College of Medicine, Bronx, NY USA; 7 Department of Medicine, Memorial Sloan-Kettering Cancer Center, New York, NY USA; 8 Cancer Dormancy Institute, Albert Einstein College of Medicine, Bronx, New York, NY USA

**Keywords:** Cancer stem cells, Cancer

Correction to: *British Journal of Cancer* 10.1038/s41416-025-03074-6, published online 24 June 2025

The PDF version of this originally published article was the uncorrected proof; it has now been replaced by the corrected version.

