# Supplementary material for: Wnt/ERK/CDK4/6 activation in the partial EMT state coordinates mammary cancer stemness with self-renewal and inhibition of differentiation
Source: Br J Cancer. 2025 Jun 24;133(7):986–1002. doi: 10.1038/s41416-025-03074-6 (PMC12480652; doi:10.1038/s41416-025-03074-6)
Supplement: Supplementary file 1 — Supplemental material [file 41416_2025_3074_MOESM1_ESM.pdf]

A

B

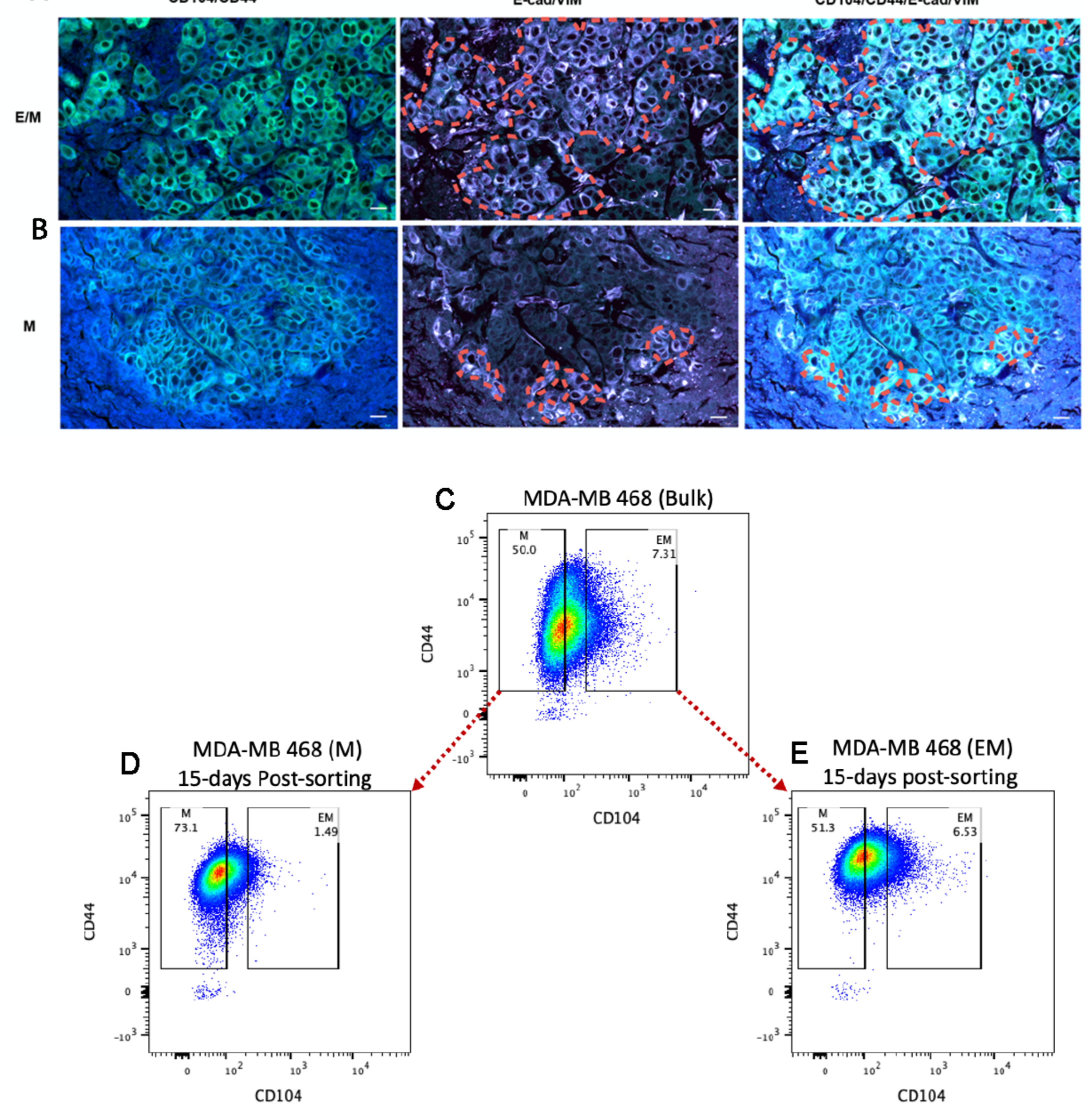

F

	E/M	M
Mice with Lung Mets	6/6	2/6
Incidence	100%	33.3%

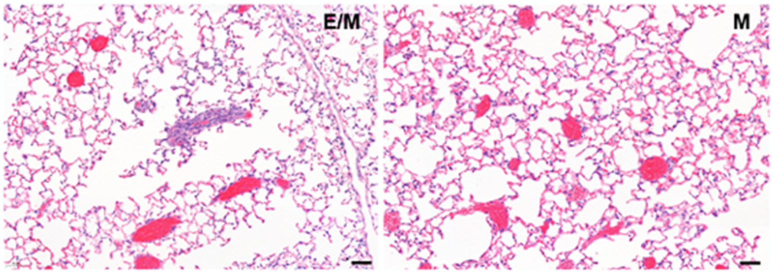

Fig. S1

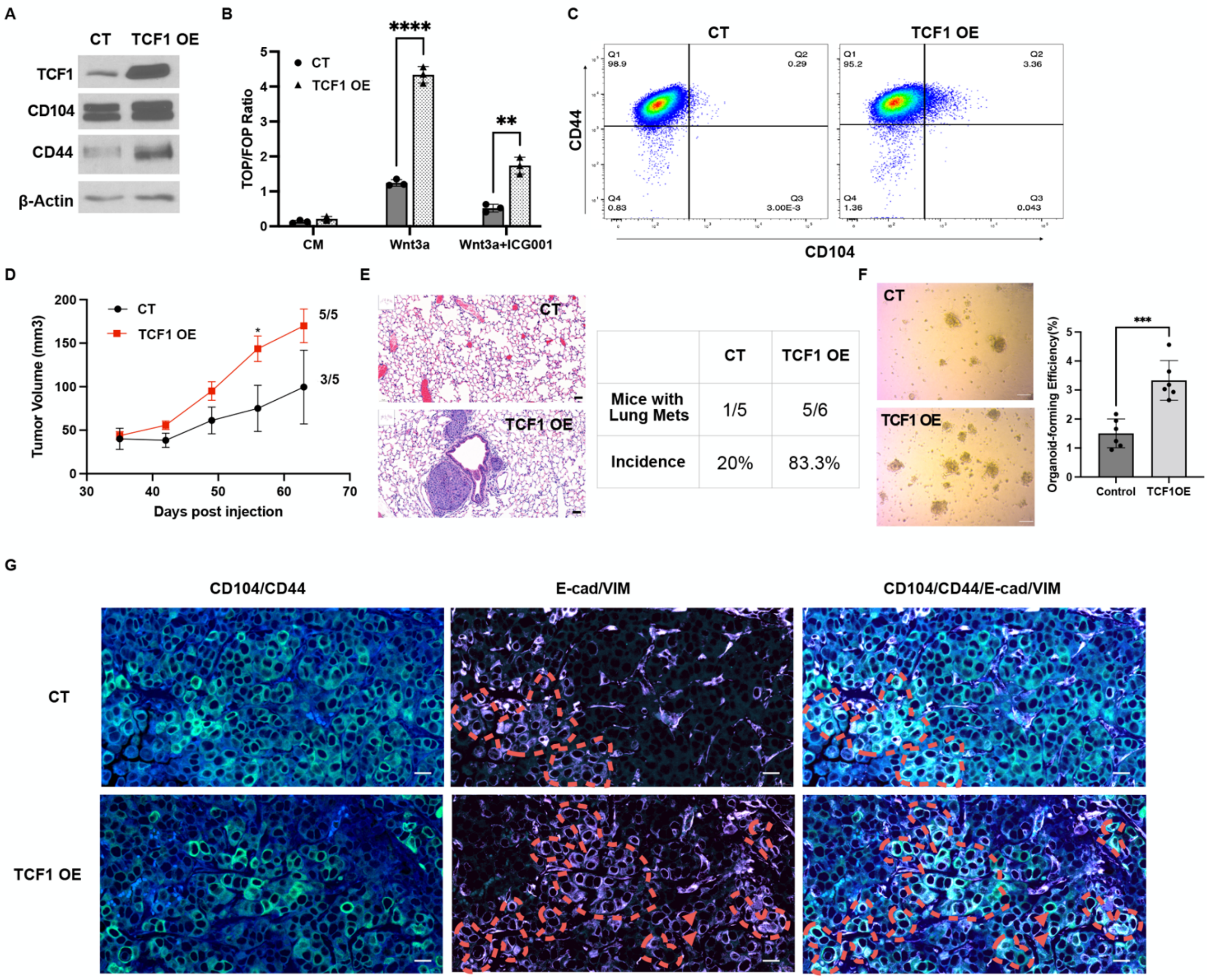
A
CT
TCF1 OE
TCF1
CD104
CD44
β-Actin
B
TOP/FOP Ratio
CT
TCF1 OE
****
**
CM
Wnt3a
Wnt3a+ICG001
C
CT
TCF1 OE
CD44
CD104
Q1 98.9
Q2 0.29
Q4 0.83
Q3 3.00E-3
Q1 95.2
Q2 3.36
Q4 1.36
Q3 0.043
D
Tumor Volume (mm3)
Days post injection
CT
TCF1 OE
5/5
3/5
E
CT
TCF1 OE
Mice with Lung Mets
1/5
5/6
Incidence
20%
83.3%
F
CT
TCF1 OE
Organoid-forming Efficiency(%)
***
Control
TCF1OE
G
CD104/CD44
E-cad/VIM
CD104/CD44/E-cad/VIM
CT
TCF1 OE

Fig. S2

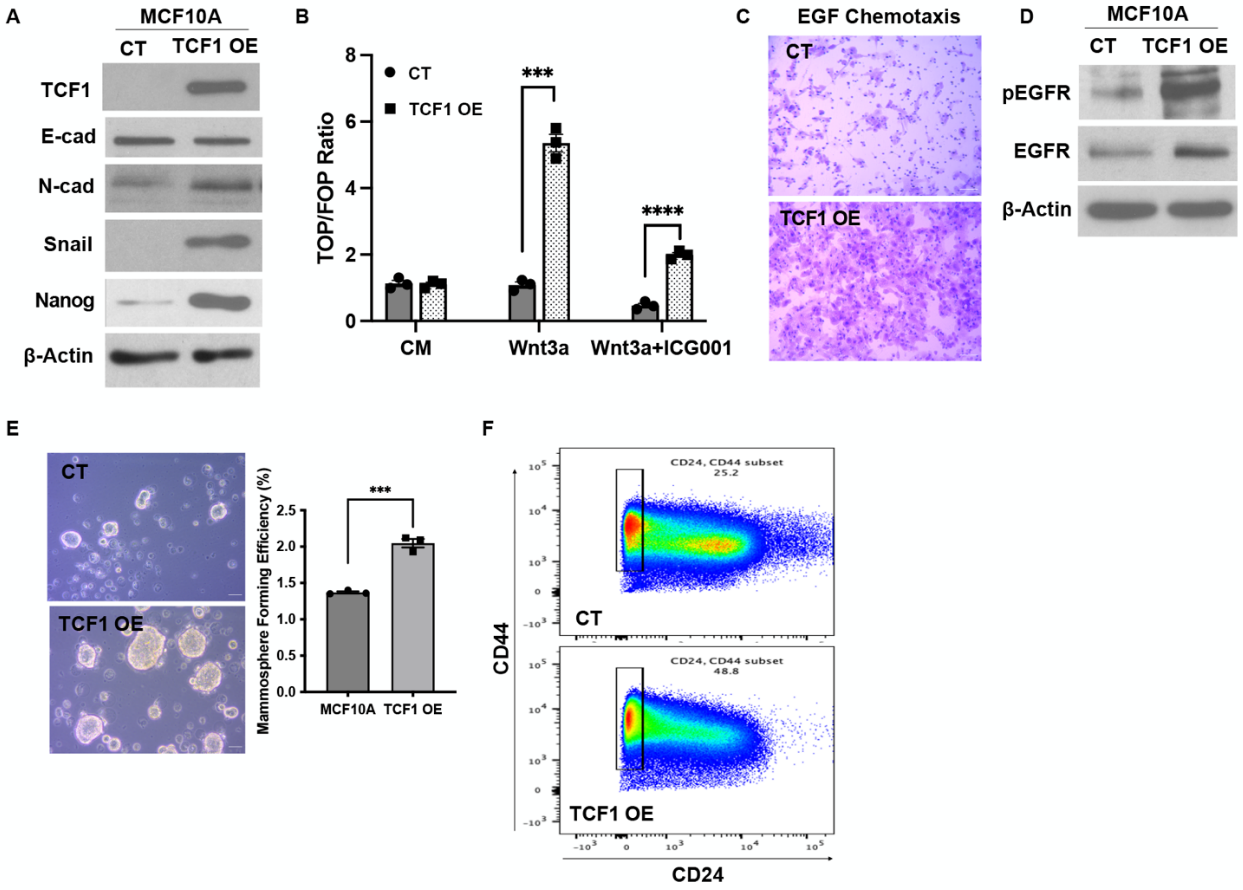

A
MCF10A
CT
TCF1 OE
TCF1
E-cad
N-cad
Snail
Nanog
β-Actin
B
TOP/FOP Ratio
CT
TCF1 OE
***
****
CM
Wnt3a
Wnt3a+ICG001
C
EGF Chemotaxis
CT
TCF1 OE
D
MCF10A
CT
TCF1 OE
pEGFR
EGFR
β-Actin
E
CT
TCF1 OE
Mammosphere Forming Efficiency (%)
***
MCF10A
TCF1 OE
F
CD24, CD44 subset
25.2
CT
CD24, CD44 subset
48.8
TCF1 OE
CD44
CD24

Fig. S3

A

MDA-MB-468:

Gene	Bulk.1	Bulk.2	E/M.1	E/M.2	M.1	M.2
RB1	21.3596201	23.1888076	22.3475096	16.8691962	23.484437	16.7213895
RBL1	898.884011	1009.21724	853.268548	970.541087	1017.34581	982.627534
RBL2	2629.90322	2063.80388	2196.15072	1826.37164	2673.46831	2159.02646

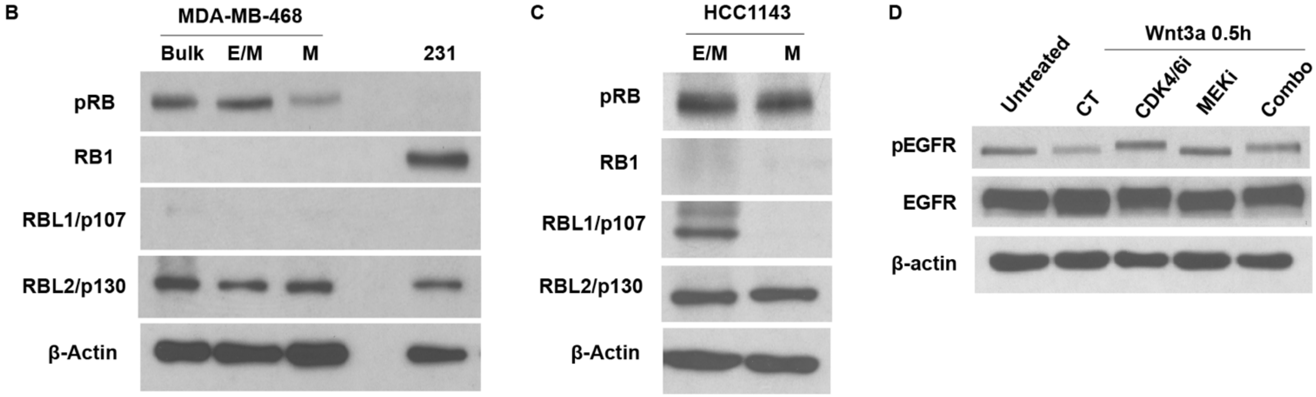

Fig. S4

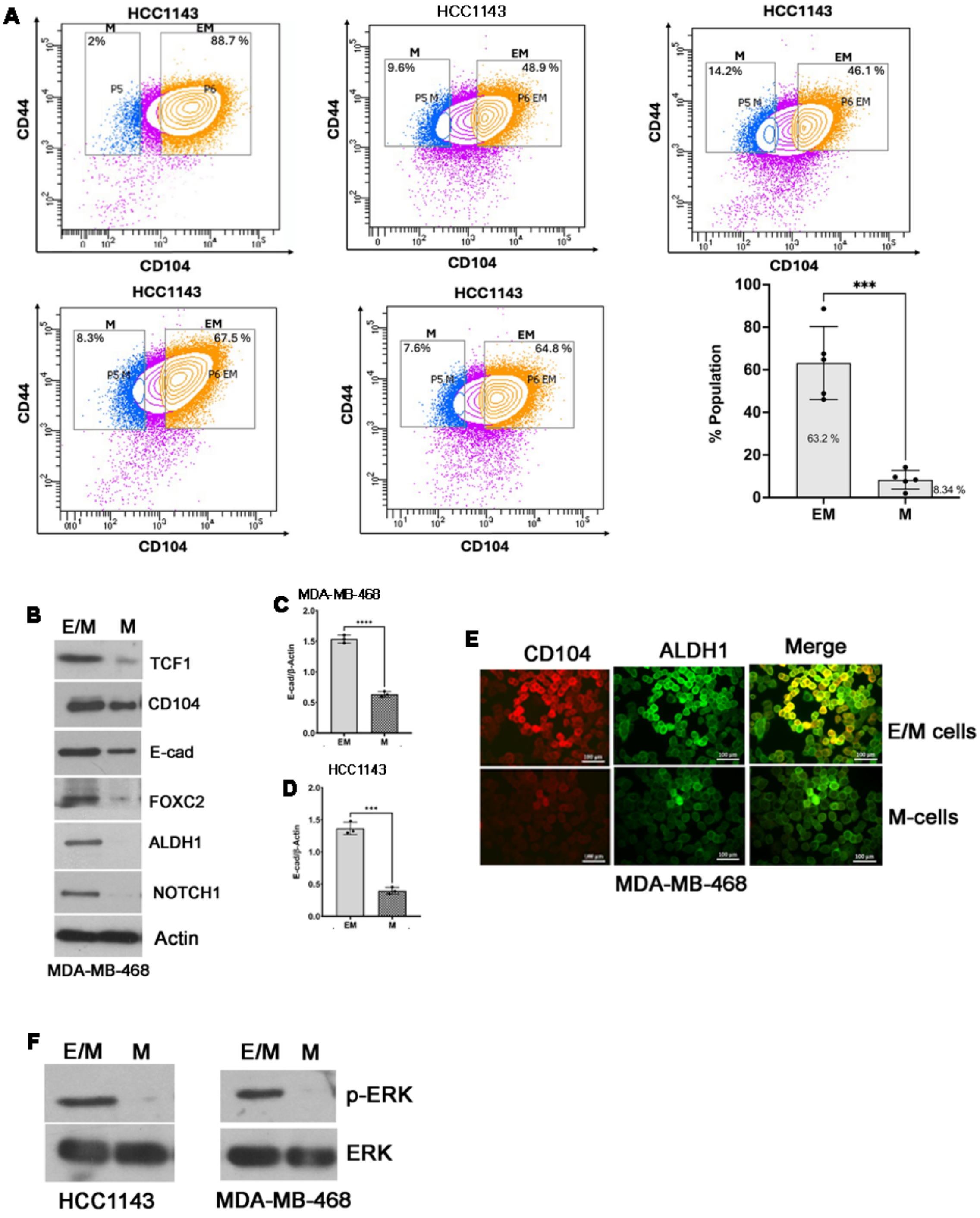
A
HCC1143
M
EM
2%
88.7 %
9.6%
48.9 %
14.2%
46.1 %
8.3%
67.5 %
7.6%
64.8 %
CD44
CD104
% Population
***
63.2 %
8.34 %
EM
M
B
E/M
M
TCF1
CD104
E-cad
FOXC2
ALDH1
NOTCH1
Actin
MDA-MB-468
C
MDA-MB-468
****
E-cad/β-Actin
D
HCC1143
***
E
CD104
ALDH1
Merge
E/M cells
M-cells
MDA-MB-468
F
p-ERK
ERK
HCC1143
MDA-MB-468

Fig. S5

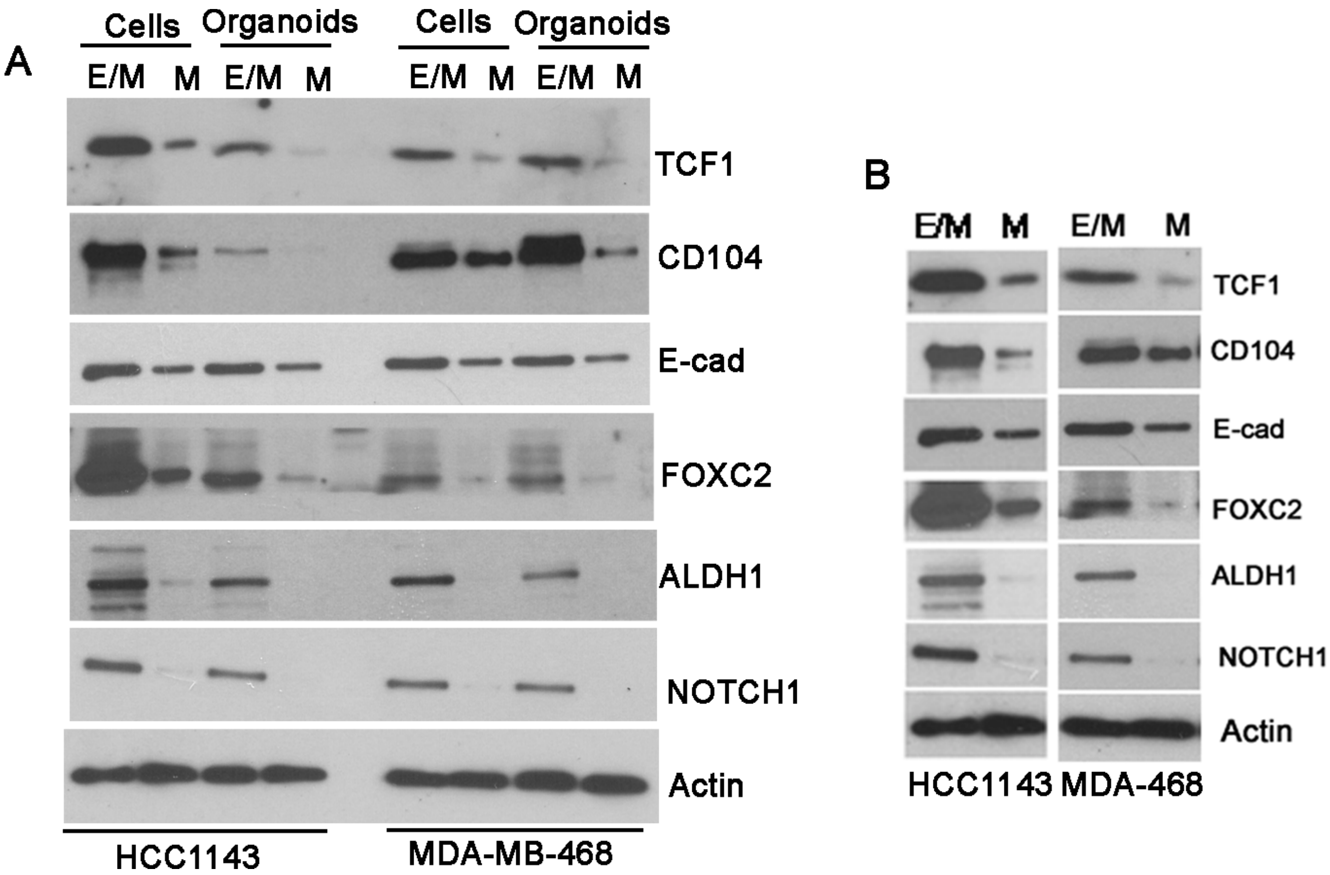
A
Cells
Organoids
Cells
Organoids
E/M
M
E/M
M
E/M
M
E/M
M
TCF1
CD104
E-cad
FOXC2
ALDH1
NOTCH1
Actin
HCC1143
MDA-MB-468
B
E/M
M
E/M
M
TCF1
CD104
E-cad
FOXC2
ALDH1
NOTCH1
Actin
HCC1143
MDA-468

Fig. S6

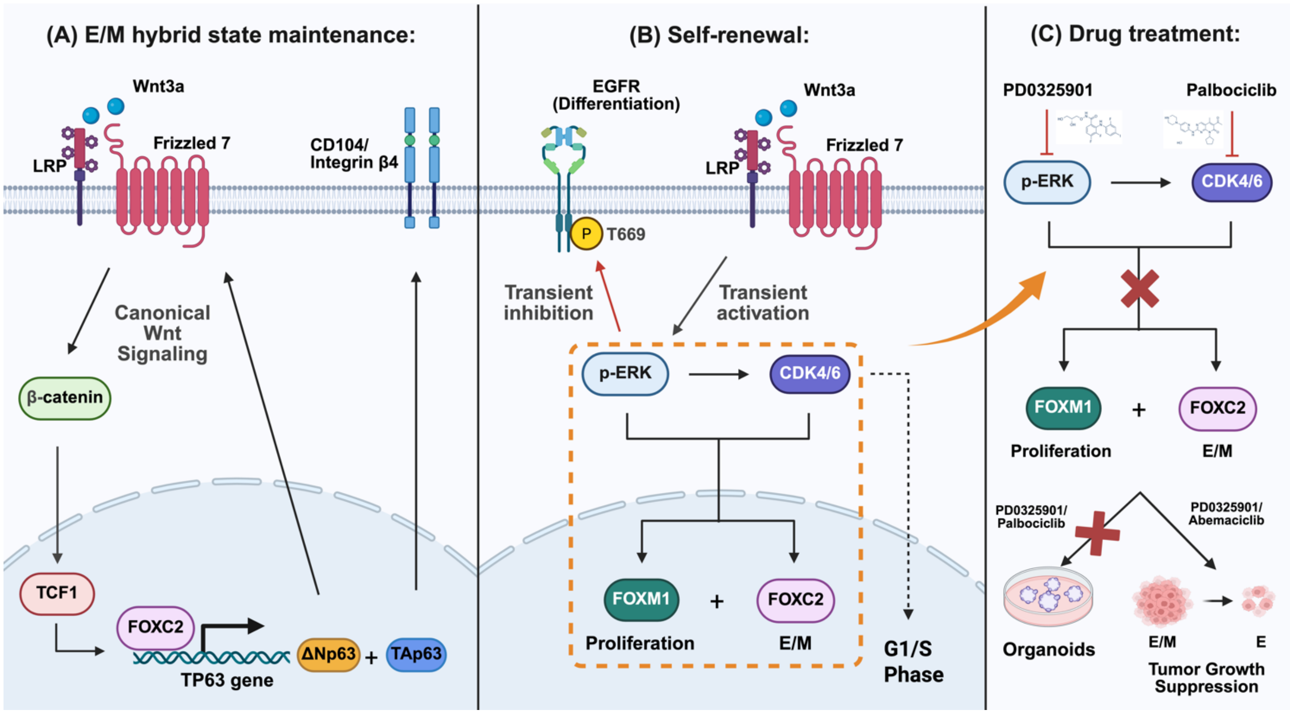
(A) E/M hybrid state maintenance:
Wnt3a
LRP
Frizzled 7
CD104/
Integrin β4
Canonical
Wnt
Signaling
β-catenin
TCF1
FOXC2
TP63 gene
ΔNp63
+
TAp63
(B) Self-renewal:
EGFR
(Differentiation)
P
T669
Wnt3a
LRP
Frizzled 7
Transient
inhibition
Transient
activation
p-ERK
CDK4/6
FOXM1
+
FOXC2
Proliferation
E/M
G1/S
Phase
(C) Drug treatment:
PD0325901
Palbociclib
p-ERK
CDK4/6
FOXM1
+
FOXC2
Proliferation
E/M
PD0325901/
Palbociclib
PD0325901/
Abemaciclib
Organoids
E/M
E
Tumor Growth
Suppression

Fig. S7

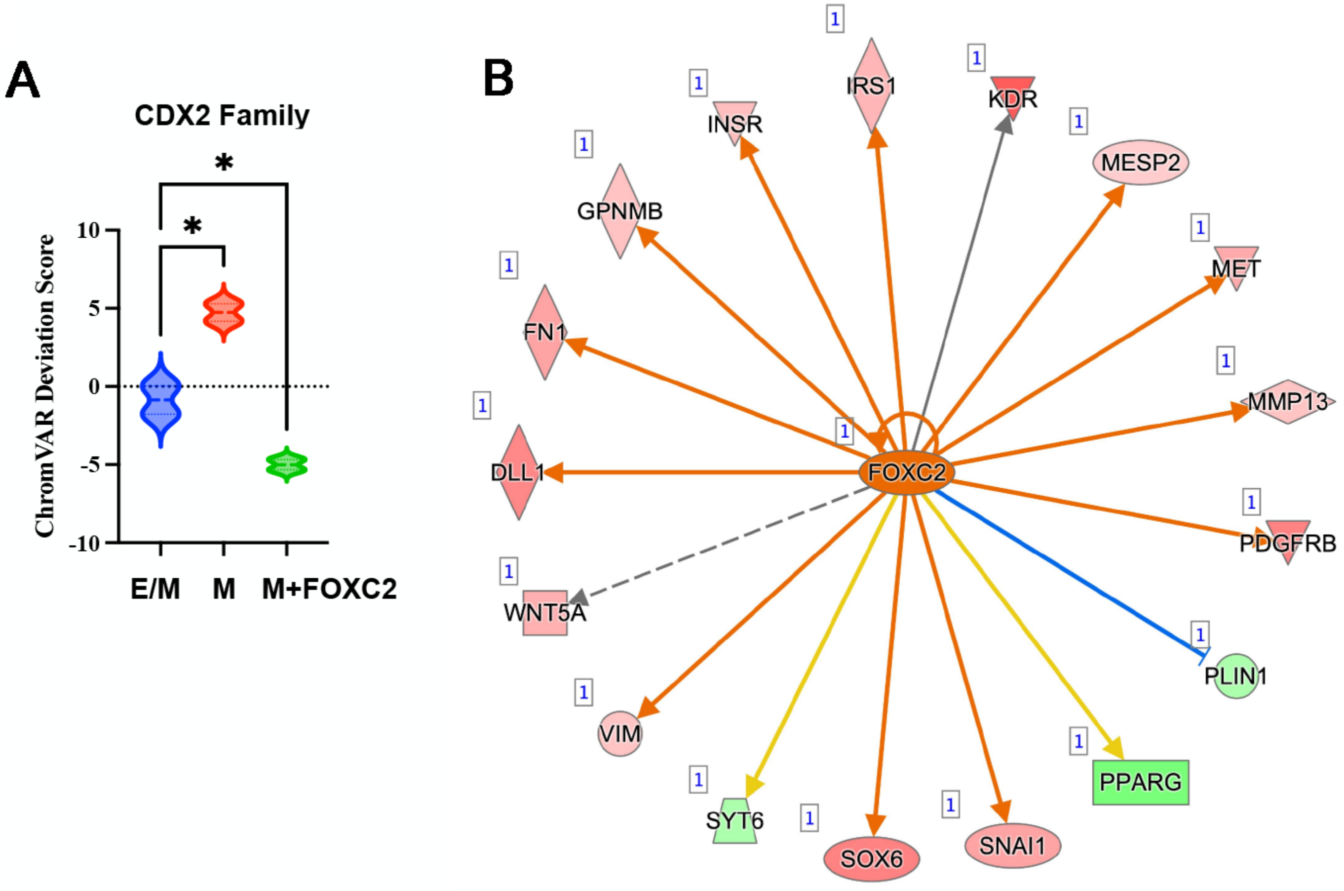

Fig.S8

Table S1. List of Oligonucleotides.

Human TAp63 sgRNA	5'-TTTGTCGCACCATCTTCTGA-3'
Human ∆Np63 sgRNA	5'- TACCTCACTAAATTGAGTCT-3'
Human ∆Np63 sgRNA	5'- TTCATATTGTAAGGGTCTCG-3'
Human TAp63 forward	5'-ATGGACTGTATCCGCATGC-3'
Human TAp63 reverse	5'-TGTTATAGGGACTGGTGGACGAG-3'
Human ∆Np63 forward	5-TACCTGGAAAACAATGCCCAG -3'
Human ∆Np63 reverse	5-TGGTCTGTGTTATAGGGA-3'
Human TAp73 forward	5'-TGATGGGGGCACCACGTTTG-3'
Human TAp73 reverse	5'- ACTGGGCCATGACAGATGTAGTCA -3'
Human ∆Np73 forward	5'- CCATGCTGTACGTCGGTGACCC-3'
Human ∆Np73 reverse	5'- TTGGAGGGGATGACAGGCGCC-3'
Human FZD7 forward	5'- GCAGCGCAAATCTGAGGTTTCCC-3'
Human FZD7 reverse	5'- GTGTGACAGCACACTGCTCTTCACT-3'

Supplemental Figures legends

Figure S1. Plasticity of the hybrid EMT phenotype *vs* metastatic potential.

(**A-B**) Staining of E/M (A) and M (B) tumors for CD104 (green), CD44 (blue), E-cad (magenta), and VIM (cyan); 20 μm. Red dashed lines indicate CD104/CD44/E-cad/VIM co-expressing areas. (**C-E**) Representative flow cytometry plots from MDA-MB-468 that were comprised of 7.3% E/M and 50% M cells (**C**). The M (**D**) or E/M (**E**) subpopulations were each separately cultured for 15 days in growth media and analyzed by flow cytometry for the percentage of E/M and M cells by CD104/CD44 cell surface expression. (**F**) Incidence of mice with lung mets post mammary fat pad injection of E/M or M cells; 6 athymic female nude mice/group are shown in table. Images from lung mets in mice that were injected with E/M or M cells are shown; 50 μm (right panels)

Figure S2. TCF1 overexpression in MDA-MB-468 cells stimulates a hybrid EMT phenotype leading to organoid formation, increased mammary tumor growth and metastasis.

(**A**) Western blots for TCF1, CD104, CD44 in control MDA-MB-468 cells and TCF7-OE cells. (**B**) TOP/FOP Flash in MDA-MB-468 control cells (circle), and TCF7 OE cells (triangle) that were untreated (CM) or treated with Wnt3a or Wnt3a+ICG001; Mean ± SEM; Unpaired t-test (**$p < 0.01$; ****$p < 0.0001$). (**C**) CD104 and CD44 FACS profiles of MDA-MB-468 control and TCF7 OE cells. (**D**) Tumor growth (volume); Mean ± SEM generated by m.f.p injection of MDA-MB-468 control and TCF7 OE cells at 7 weeks post-injection; 5 female athymic nude mice/group, Unpaired t-test (*$p< 0.05$). (**E**) H&E-stained lung metastatic foci from mice bearing MDA-MB-468 control or TCF7 OE tumors. 5 mice/group, scale bar: 50 μm. Table shows incidence of mice with spontaneous lung metastasis 12 weeks post-surgical excision of primary tumor at 1cm diameter. (**F**) Representative organoids formed by MDA-MB-468 control or TCF7 OE tumor cells. N=3, scale bar: 100 μm. Unpaired t-test; Mean ± SEM (***$p < 0.001$). (**G**) Immunostaining of CD104 (Green) and CD44 (Blue), E-cad (Magenta), and VIM (Cyan) on MDA-MB-468 control and TCF7 OE mammary tumors. Scale bar: 20 μm. Red dashed lines indicate CD104/CD44/E-cad/VIM co-expressing areas.

Figure S3. TCF1 overexpression in normal MCF10A cells stimulates a hybrid EMT state.

(**A**) Immunoblotting (IB) of MCF10A and TCF1 OE cell lines for indicated EMT proteins. (**B**) TOP/FOP Flash activity in control MCF10A (circle), and TCF1 OE (square) cells, untreated (CM) or treated with Wnt3a or

Wnt3a+ICG001; Mean ± SEM; Unpaired t-test (***p < 0.001; ****p < 0.0001). (**C**) Images of transmigrated MCF10A control and TCF1 OE cells, scale bar: 100 μm. (**D**) IB of p-EGFR (Y1173) and EGFR in MCF10A control and TCF1 OE cells. (**E**) Images of MCF10A control and TCF1 OE mammospheres, scale bar: 100 μm; Mean ± SEM; *** p < 0.001. (**F**) CD24 and CD44 FACS profiles of MCF10A control and TCF7 OE cells.

Figure S4. RBL1 and RBL2 expression in MDA-MB-468 and HCC1143 cell lines.

(**A**) Normalized RB1, RBL1, and RBL2 mRNA expression in MDA-MB-468 bulk, E/M, and M cells from RNA-sequencing. (**B**) IB of RB1, RBL1, RBL2, pRb, in MDA-MB-468 bulk, E/M, and M cells. MDA-MB-231 (231) is used as positive control. (**C**) IB of RB1, RBL1, RBL2, pRb in HCC1143 E/M, and M cells. (**D**) IB of pEGFR (Y1173) and EGFR in MDA-MB-468 E/M cells treated with vehicle, 10 μM CDK4/6i (palbociclib), 1 μM MEKi (PD901), or both (combo) for 24 hrs, followed by addition of 100 ng/ml Wnt3a in 0.2%FBS/RPMI for 30 min.

Figure S5. The hybrid EMT state in HCC1143 and MDA-MB-468 TNBC cells.

(**A**) CD104/CD44 flow cytometry charts using HCC1143 cells in 5 independent experiments. The percentage of E/M vs M is shown as Mean ± SEM; Unpaired t-test; ***p < 0.001. (**B**) E/M and M subpopulations from MDA-MB-468 were immunoblotted for TCF1, CD104, E-cad, FOXC2, ALDH1, NOTCH1 vs Actin. (**C-D**) E-cad immunoblots in HCC1143 (C) and MDA-MB-468 (D) 3 independent experiments were quantified by densitometry; Mean ± SEM; Unpaired t-test; ****p < 0.001, ***p < 0.001. (**E**) MDA-MB-468 E/M cells (top panels) and M cells (bottom panels) were co-immunostained with CD104 (TRITC) (right) and ALDH1 (FITC) (middle) and CD104/ALDH1 is shown in the merged panel (right). (**F**) E/M and M cells from HCC1143 (left) or MDA-MB-468 (right) were immunoblotted for p-ERK or ERK.

Figure S6. Hybrid EMT markers in HCC1143 and MDA-MB-468 cells and organoids.

(**A**) HCC1143 and MDA-MB-468 cells grown in culture or as organoids were lysed and immunoblotted on the same gel for TCF1, CD104, E-cad, FOXC2, ALDH1, NOTCH1 vs Actin. (**B**) A comparison of TCF1, CD104, E-cad, FOXC2, ALDH1, NOTCH1 vs Actin in HCC1143 and MDA-MB-468 cells is shown. These two panels are the same shown in Fig. 7C (HCC1143) and Fig. S5B (MDA-MB-468).

Figure S7. A model for the TCF1/ERK/CDK4/6 mediated regulation of the hybrid EMT state and impact for therapeutic translation in TNBC.

(**A**) **E/M hybrid state maintenance**: Wnt3a activates Wnt/β-catenin signaling in E/M basal-like breast cancer cells, resulting in TCF1 upregulation, which in turn upregulates FOXC2, leading to transcriptional activation of ΔNp63 and TAp63 isoforms. ΔNp63, in turn, potentiates Wnt/β-catenin signaling via Frizzled 7 upregulation, whereas TAp63 regulates CD104 expression, thereby promoting the partial EMT state. (**B**) **E/M cancer stem cell self-renewal**: Wnt3a stimulates transient ERK phosphorylation (p-ERK), which in turn stimulates CDK4/6 activation, causing S-phase transition. In parallel, transient p-ERK inactivates EGFR via threonine 669 (T669) phosphorylation on the transmembrane domain, thereby preventing EGF from promoting sustained ERK phosphorylation that causes E/M to M differentiation. In parallel, ERK/CDK4/6 upregulates FOXC2 to maintain the E/M stem-like state, and FOXM1 to drive cell cycle progression leading to self-renewal. (**C**) **CSC therapy by ERK/CDK4/6 inhibition results in E/M to E differentiation and suppression of mammary tumor growth**: Drugs that inhibit ERK using the MEK1 inhibitor PD0325901, and CDK4/6 using Palbociclib or Abemaciclib are powerful in suppressing organoid formation by E/M mammary tumor cells pre- and post-organoid formation. These drugs caused E/M to E differentiation and suppression of mammary tumor growth.

Figure S8. ATAC sequencing analysis of E/M, M, and FOXC2 overexpressing MDA-MB-468 cells.

(**A**) Violin plot of CDX2 TF family chromatin accessibility in MDA-MB-468 E/M, M cells, and FOXC2 OE M cells; $^*p<0.05$. (**B**) Upstream regulator analysis by Ingenuity Pathway Analysis comparing RNA-sequencing datasets between MDA-MB-468 E/M and M cells on FOXC2 showing a set of genes positively (pink) and negatively (green) regulated by FOXC2 in E/M cells.
